# Piezoelectric Nanomaterial‐Mediated Physical Signals Regulate Cell Differentiation for Regenerative Medicine

**DOI:** 10.1002/smsc.202300255

**Published:** 2024-01-08

**Authors:** He Li, Xueting Pan, Tianyun Wang, Zhenlin Fan, Hai Wang, Wenjie Ren

**Affiliations:** ^1^ Institutes of Health Central Plain, Clinical Medical Center of Tissue Engineering and Regeneration Xinxiang Medical University Xinxiang 453003 China; ^2^ CAS Key Laboratory for Biomedical Effects of Nanomaterials & Nanosafety CAS Center for Excellence in Nanoscience National Center for Nanoscience and Technology Beijing 100190 China; ^3^ Xinxiang University Xinxiang Henan Province 453000 China; ^4^ School of Nanoscience and Engineering University of Chinese Academy of Sciences Beijing 100049 China; ^5^ Department of Orthopedics The First Affiliated Hospital of Xinxiang Medical University Weihui 453100 Henan Province China

**Keywords:** cell differentiation, physical signaling regulation, piezoelectric nanomaterials, tissue regeneration

## Abstract

Tissue damage often causes considerable suffering to patients due to slow recovery and poor prognosis. The use of electroactive materials to deliver biophysical signals plays a key role in regulating tissue regeneration processes. Among these materials, piezoelectric materials have unique electromechanical conversion capabilities, making them suitable for use as cell scaffolds. They can deform and emit electrical signals in response to external stimuli, thereby regulating cell proliferation and differentiation. In this review, recent advances are presented in piezoelectric materials as physical signaling mediators that regulate cell differentiation. The basic mechanisms, classification of these materials, and their different applications in tissue regeneration are described. Finally, a comprehensive discussion of current challenges and prospects in the field is provided. Together, existing experimental results basically show that piezoelectric materials can improve the process and effect of tissue repair, providing new technical options for the development of tissue engineering in the future.

## Graphical abstract

In this review, research progress on the use of piezoelectric materials to regulate cell differentiation through physical signals is outlined. The mechanisms behind physical signal‐mediated cell differentiation and its applications in tissue regeneration are discussed. The review also provides an in‐depth discussion of current challenges and prospects in the field.

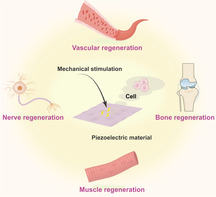

## 1 Introduction

Organisms have inherent self‐repair mechanisms, but some natural cells exhibit limited regenerative capabilities in the face of severe trauma and neurodegenerative diseases.^[^ ^1^, ^2^ ^]^ This is particularly evident in nondividing cells, such as nerve cells and skeletal muscle cells, which lack the ability to divide and regenerate postnatally.^[^ ^3^, ^4^ ^]^ Consequently, relying solely on autologous repair capabilities is challenging in addressing extensive tissue damage. Traditional treatments such as tissue and organ transplants face limitations due to donor shortages and the risk of immune rejection.^[^ ^5^, ^6^ ^]^ Tissue engineering is a promising therapeutic strategy for tissue regeneration, consisting of three elements: scaffolds, cells, and growth factors.^[^ ^7^, ^8^, ^9^, ^10^, ^11^ ^]^ Cells play a crucial role in tissue engineering, and inducing cell differentiation is an important way to obtain certain types of cells for tissue repair. The differentiation potential of stem cells renders them highly favored in the research field of tissue and organ injury repair. Stem cells can be divided into embryonic stem cells, adult stem cells, and induced pluripotent stem cells according to their differentiation capabilities.^[^ ^12^ ^]^ In clinical research and applications, people tend to use adult stem cells and induced pluripotent stem cells to avoid the ethical issues related to embryonic stem cells.^[^ ^13^ ^]^ Currently, a variety of methods to regulate cell differentiation have been explored, including but not limited to mechanical stimulation, light, electrical stimulation, thermal factors, and pharmaceutical intervention.^[^ ^14^ ^]^ Among them, electrical stimulation is best known for its ability to modulate cell differentiation.^[^ ^15^, ^16^ ^]^ Electrical stimulation is an integral component of maintaining normal biological activities in living organisms. Especially in excitable tissues, Na^+^ and K^+^ ion‐mediated action potentials (ranging from −10 to −90 mV) can trigger the proliferation and differentiation of different types of cells through intercellular communication.^[^ ^17^, ^18^ ^]^ Electric fields play a crucial role in cell physiology, regulating most cellular processes in nearly all cell types. Therefore, generating and modulating electrical signals shows great potential for rapid recovery of injured tissue.^[^ ^19^ ^]^ In view of this, electroactive nanomaterials with excellent electrical and mechanical properties have been developed.^[^ ^20^, ^21^, ^22^ ^]^

Electroactive nanomaterials are generally divided into two categories: conductive nanomaterials and nonconductive nanomaterials. Piezoelectric nanomaterials belong to the category of nonconductive nanomaterials and are widely used in many fields such as antimicrobial,^[^ ^23^, ^24^ ^]^ tumor treatment,^[^ ^25^ ^]^ and biosensing.^[^ ^26^, ^27^ ^]^ Interestingly, traces of naturally occurring piezoelectric materials can be found in components of the human body such as bones, skin, amino acids, and proteins.^[^ ^28^, ^29^, ^30^, ^31^ ^]^ These natural biological materials have brought new ideas to the research of piezoelectric materials. To generate electrical signals, stimulation signals mediated by piezoelectric materials can be implemented in a noninvasive manner. Relying on the natural properties of the material, it forms mechanical deformations in response to external stimuli and can generate alternating electric fields. At the same time, as a medium for cell growth, their surface nanostructures provide dual intervention for cell growth and differentiation. Some developed piezoelectric materials, such as barium titanate (BaTiO_3_), zinc oxide (ZnO), potassium sodium niobate (KNN), and polyvinylidene fluoride (PVDF), have simple structures, definite compositions, small size, controllable morphology, and high biocompatibility.^[^ ^32^, ^33^, ^34^ ^]^ Unlike most dynamic therapies based on energy conversion, piezoelectric nanomaterials do not require extensive external energy input and related equipment during the treatment process. It can utilize mechanical energy generated by external ultrasonic vibration or physiological movements and convert it into electrical energy through its unique internal structure.^[^ ^35^, ^36^ ^]^ This highly efficient sensing and conversion capability not only reduces treatment costs but also reduces the potential risk of injury or infection that may occur during surgery.^[^ ^37^, ^38^, ^39^, ^40^ ^]^ The distinctive features of local noninvasive treatment significantly enhance clinical applicability.

**Scheme 1:**
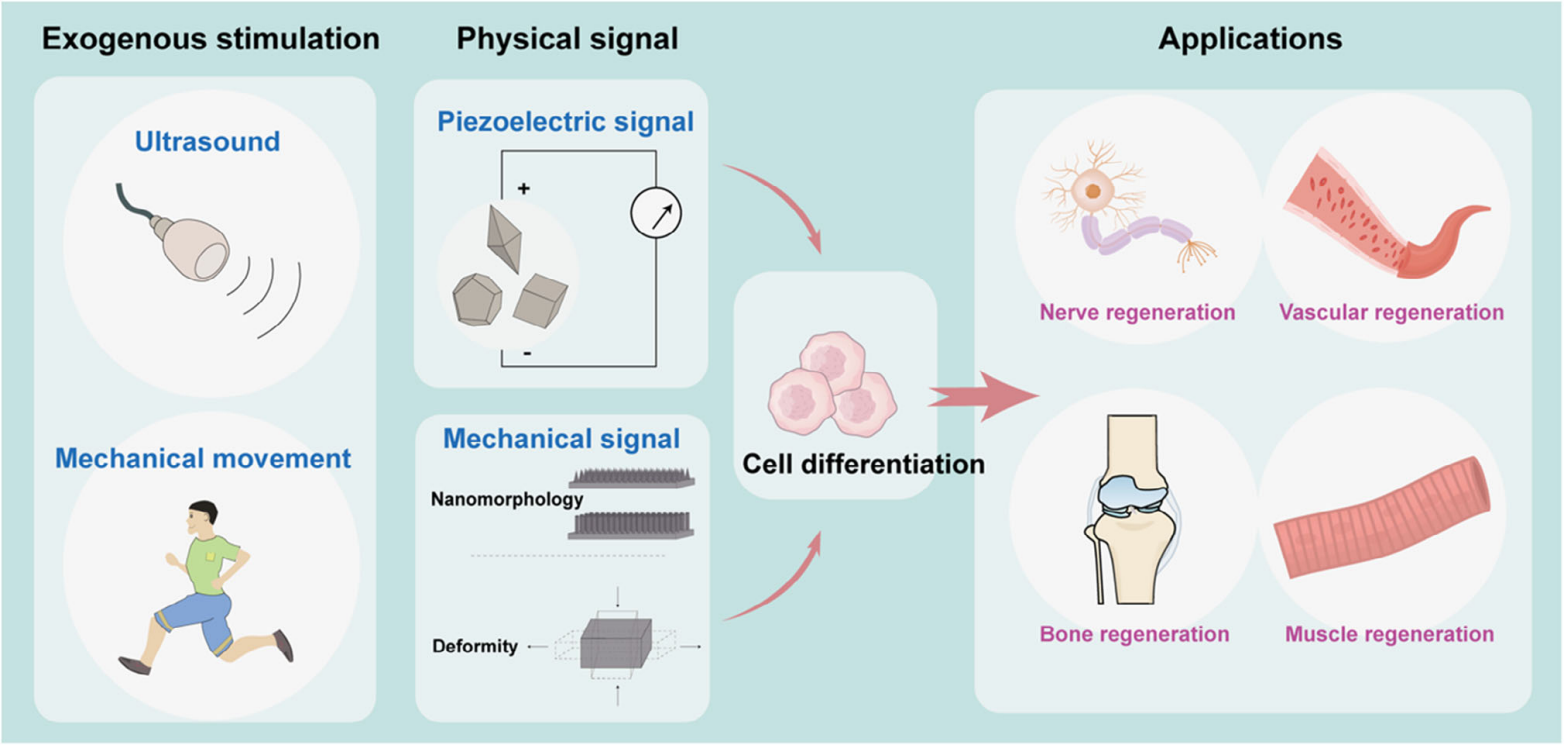
Piezoelectric nanomaterial‐mediated physical signaling regulates cell differentiation to promote tissue regeneration.

In recent years, due to the increasing research on electrical stimulation in the field of cell differentiation, many research teams have turned their attention to this emerging field. Despite the popularity of reviews on piezoelectric nanomaterials, the field of cell differentiation still lacks a systematic summary. This article is dedicated to a comprehensive review of studies involving the use of piezoelectric nanomaterials to induce cell differentiation (**Scheme** **1**). We begin with an overview of piezoelectric nanomaterials and subsequently introduce the mechanisms by which physical signals based on piezoelectric nanomaterials stimulate cell differentiation. Next, we summarize the research and applications related to cell differentiation in various diseases, including nerve regeneration, bone regeneration, muscle regeneration, and vascular regeneration. Finally, we discuss current challenges and opportunities in this field. This review aims to provide some assistance to the development of this emerging field.

## 2 Piezoelectric Nanomaterials

### 2.1 Overview of Piezoelectric Materials

#### 2.1.1 Piezoelectric Mechanisms

Piezoelectric materials are a unique class of materials that can convert mechanical energy into electrical energy and vice versa.^[^ ^41^ ^]^ Piezoelectricity results from noncentrosymmetric properties of materials, such as the asymmetric structure of inorganic piezoelectric crystals and the molecular structure and orientation of organic piezoelectric polymers. In inorganic piezoelectric materials, the generation of piezoelectricity is attributed to the movement of ions within the crystal structure. When a crystal is subjected to mechanical stress, the atomic structure within the crystal changes, resulting in the displacement of positive and negative ions within the structure and subsequent generation of a dipole moment.^[^ ^42^ ^]^ The inherent asymmetric central structure of piezoelectric materials ensures that the dipole formed is not canceled by other dipoles within the crystal unit. BaTiO_3_ nanocrystals are one of the most widely studied inorganic piezoelectric materials and can be synthesized by various chemical methods such as organometallic method, solvothermal/hydrothermal method, template method, molten salt method, and sol–gel method.^[^ ^43^ ^]^ In the octahedral unit cell of BaTiO_3_, the Ti^4+^ ions move slightly upward from the center of the spatial unit cell relative to the O^2−^ ions, creating an inherent asymmetry that leads to the spontaneous formation of polarization. BaTiO_3_ has high dielectric constant, ferroelectricity and piezoelectricity. These properties have broad utility in applications such as nonvolatile random access memory, nonlinear optics, and more.^[^ ^44^ ^]^ However, for some nonferroelectric piezoelectric materials, such as ZnO, electrodeposition does not manifest itself unless the crystal lattice is mechanically deformed.^[^ ^45^ ^]^ In organic materials, piezoelectricity arises through the reorientation of molecular dipoles.^[^ ^46^ ^]^ PVDF is a representative of organic piezoelectric materials with different crystalline forms (*α, β, γ, δ, ε*), of which the *α* phase and *β* phase are the most commonly used. The *α* phase is thermodynamically stable at 298 K and 1 bar but lacks piezoelectric properties. In contrast, the *β* phase exhibits excellent piezoelectric properties, which is mainly attributed to the arrangement of highly electronegative fluorine atoms on the same side of the carbon chain.^[^ ^47^, ^48^, ^49^, ^50^ ^]^

In addition, the discovery of endogenous biological materials with piezoelectric properties in living organisms has greatly advanced the exploration of piezoelectric materials. Amino acids are the basic components of proteins and mainly exist in l‐ or d‐type chiral symmetry groups.^[^ ^51^ ^]^ Of the 20 amino acids that make up human proteins, 17 of them and their compounds exhibit piezoelectricity (except glutamine, phenylalanine, and tryptophan).^[^ ^52^ ^]^ Glycine is the only achiral amino acid among the 20 amino acids and has three crystal forms: *α, β*, and *γ*. It is worth noting that *β*‐glycine and *γ*‐glycine have strong piezoelectric properties due to their crystal noncentrosymmetric point clusters.^[^ ^53^, ^54^, ^55^ ^]^ Proteins with piezoelectric properties, such as collagen and silk proteins, are also used in biotherapeutics and related fields.^[^ ^56^, ^57^ ^]^ Filipin proteins consist of a heavy chain (≈370 kDa) and a light chain (approximately 26 kDa) linked by disulfide bonds. Filipin proteins exhibit crystal polymorphism, with filipin I crystals being orthorhombic and filipin II crystals having monoclinic single crystal units. Both crystal units lack a center of symmetry, indicating that filipin proteins have piezoelectric properties.^[^ ^58^, ^59^ ^]^ Various tissues and organs such as bones, skin, tendons, and sclera also exhibit piezoelectric properties.^[^ ^60^, ^61^ ^]^ For example, the piezoelectricity of bone is attributed to collagen molecules with a noncentrosymmetric structure.^[^ ^62^ ^]^ Among the composition of bone, hydroxyapatite, which constitutes the major content, is a ceramic material with a centrosymmetric structure and lacks ferroelectric or piezoelectric properties. However, studies have shown that hydroxyapatite exhibits electronegativity at low sintering temperatures.^[^ ^21^ ^]^ Thus, the electrical activity in bones is thought to be the result of a combination of collagen and hydroxyapatite. During exercise, the mechanical forces generated on bones continuously trigger electrical signals, gradually promoting bone growth and remodeling.

**Figure 1:**
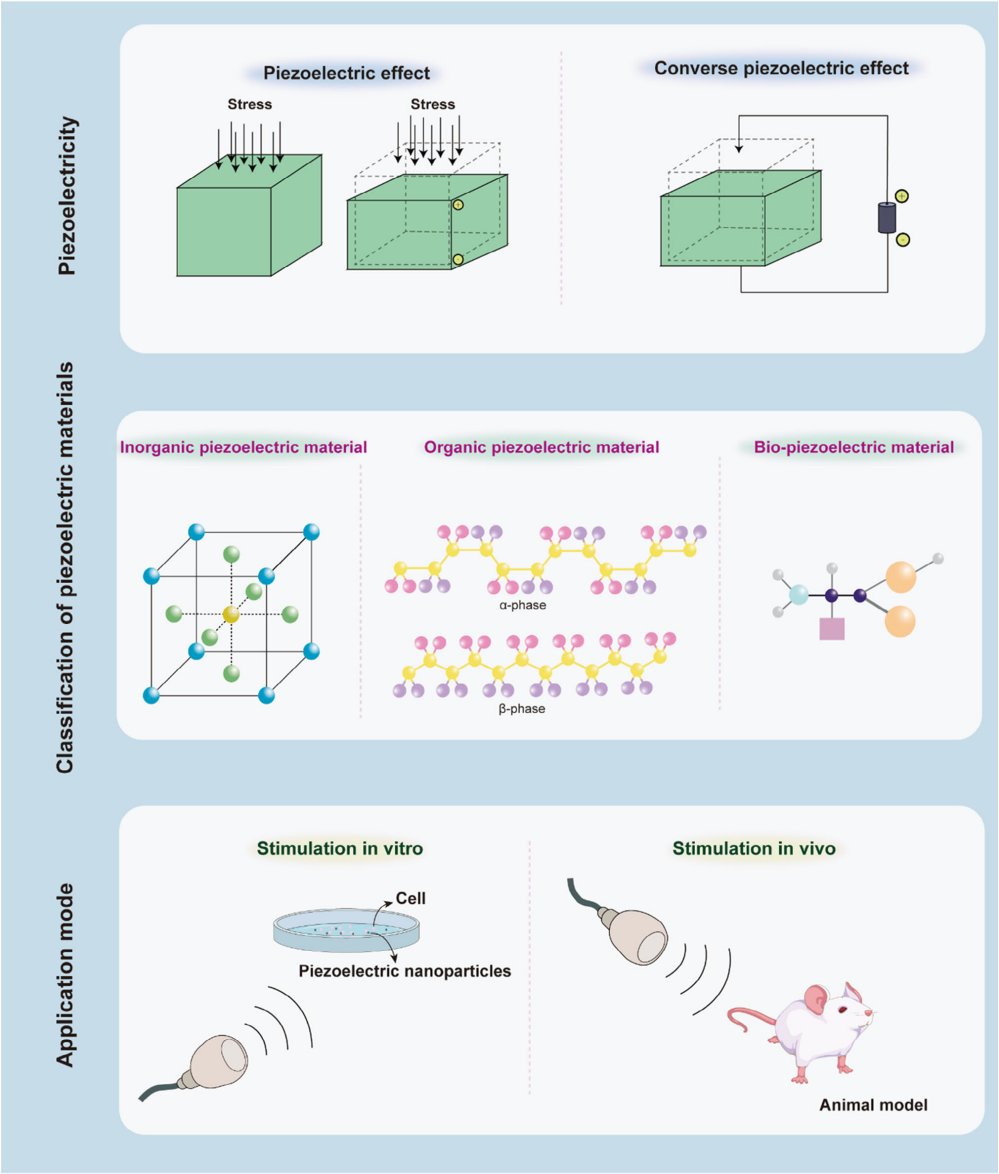
Schematic illustration of the piezoelectric properties, classification, and applications of piezoelectric materials.

In biomedical applications, ultrasonic waves or biological activity are often used to activate piezoelectric materials to achieve the desired purpose.^[^ ^63^, ^64^, ^65^, ^66^ ^]^ Ultrasonic waves refer to sound waves with a frequency exceeding 20 000 Hz, which are characterized by good directionality, high sound energy transmission rate, and strong penetrating ability.^[^ ^67^ ^]^ When ultrasound interacts with the medium, it causes a mechanical effect, which subsequently activates the piezoelectric materials to produce electrical signals, thereby promoting cell differentiation and tissue regeneration.^[^ ^46^, ^68^ ^]^ Similarly, human body activities, such as trunk movement, blinking, heartbeat, and so on, can provide abundant kinetic energy for the activation of piezoelectric materials. In addition, studies have shown that deformation of piezoelectric materials through cell pulling is also a good method of piezoelectric stimulation.^[^ ^69^ ^]^ **Figure** **1** depicts the general concept of piezoelectric materials.

#### 2.1.2 Quantification of Piezoelectric Effect

Testing precise piezoelectric effect at the nanoscale is challenging. Since 1992, with the continuous exploration of piezoelectric response force microscopy (PFM), the piezoelectric properties of nanomaterials have been quantitatively studied.^[^ ^70^ ^]^ PFM is widely used in the characterization of nanoscale piezoelectric materials, and its basic principle depends on the application of the inverse piezoelectric effect of materials.^[^ ^71^, ^72^ ^]^ This involves pressing the conductive tip of the PFM against the sample surface and inducing periodic deformation of the sample by applying an oscillating electric field to the tip. This deformation is then transferred to the cantilever oscillations and detected by a quadrant photodetector using phase‐locking technology. The piezoelectric coefficient is represented as *d*_*ij*_, where *d* corresponds to the piezoelectric coefficient tensor, and *i* and *j* represent the direction of polarization and the direction of applied stress, respectively.^[^ ^46^ ^]^ For example, the piezoelectric coefficient *d*_33_ characterizes the material's ability to generate charge when strain is applied perpendicular to the strain plane and is a key factor in characterizing the axial/in‐plane piezoelectricity of nanofilms and nanotubes/wires.^[^ ^73^ ^]^ The numerical magnitude is usually related to nanoscale geometry, crystal orientation, and temperature.^[^ ^74^, ^75^ ^]^ The higher the *d* value, the material produces higher voltage output under the same deformation. Stretching, shearing, bending, or compressing a piezoelectric material creates a piezoelectric potential. The relative movement of positive and negative charge centers during deformation leads to polarization but does not affect the overall electrical neutrality of the material.^[^ ^76^ ^]^ However, there is currently no truly accurate method to measure the piezoelectric coefficient of nanomaterials, which is mainly due to the interference caused by the presence of surface charges on nanomaterials and their small size and smooth surface, which affects the accuracy of the measurement.

### 2.2 Mechanisms of Cell Differentiation Induced by Piezoelectric Materials

#### 2.2.1 Electrical Signal

**Figure 2:**
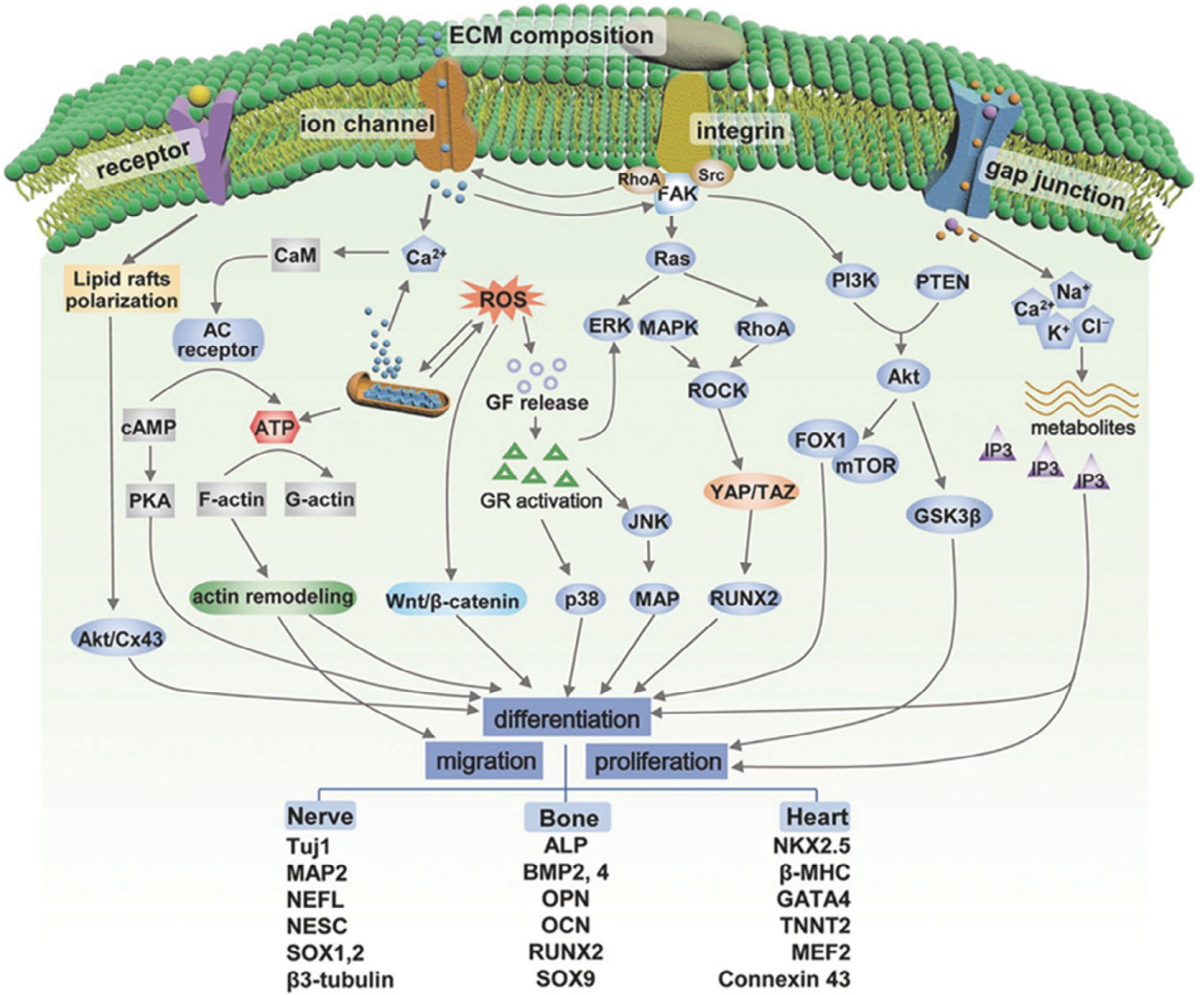
Possible pathways for electrical stimulation to induce biological responses. Reproduced with permission.^[^ ^22^ ^]^ Copyright 2021, WILEY‐VCH.

Although electrical stimulation has been recognized as an important modality to regulate cell biological behavior, cellular responses to electrical stimulation are complex and the underlying mechanisms have not been fully studied (**Figure** **2**). According to current findings, calcium ion (Ca^2+^) signaling and membrane receptors play pivotal roles in cell differentiation.^[^ ^77^, ^78^, ^79^ ^]^ 1) Ca^2+^ signaling: Ca^2+^ signaling represents one of the most versatile intracellular signaling pathways, mediating a variety of cellular processes, including apoptosis, migration, proliferation, and differentiation. In addition, electrical stimulation induces membrane protein reorganization via phospholipase C, thereby triggering the release of Ca^2+^ from intracellular calcium stores. Intracellular Ca^2+^ binds to calmodulin (CaM). CaM serves as a calcium sensor and signal transducer, binding to and activating target proteins that cannot bind Ca^2+^, thereby regulating cellular functions.^[^ ^80^ ^]^ Chen's team found that the use of ultrasound‐mediated release of electrical signals from PVDF to stimulate the differentiation of rat pheochromocytoma cells (PC12) may be related to this mechanism.^[^ ^81^ ^]^ Increased intracellular Ca^2+^ concentration can bind to CaM and subsequently activate adenylyl cyclase, which, in turn, induces neurogenesis via a cyclic adenosine monophosphate (cAMP)‐dependent pathway. Similarly, another research group induced odontogenic differentiation of dental pulp stem cells (DPSCs) by increasing Ca^2+^ unitransporter levels via electrical stimulation, resulting in increased mitochondrial Ca^2+^ levels, increased adenosine triphosphate (ATP) production, and activation of the cAMP‐protein kinase A pathway.^[^ ^40^ ^]^ 2) Membrane receptors: Membrane receptors act as signal integrators and regulate many cellular processes through ligand–receptor binding. Under electrical stimulation, ligand–receptor interactions can be modulated by altering the distribution and conformation of receptors on the membrane surface, thereby regulating intracellular signaling pathways. For example, cascade activation of mitogen‐activated protein kinase is considered a classical pathway for regulating cell fate. The study showed that surface charge on the nanocomplex BaTiO_3_/poly(vinylidene fluoride–trifluoroethylene) (BaTiO_3_/P(VDF–TrFE)) could promote the neural differentiation of human deciduous tooth stem cells (SHED) in a dose‐dependent manner.^[^ ^82^ ^]^ This phenomenon may be related to the focal adhesion kinase mechanosensing signaling pathway. In addition to Ca^2+^ signaling and membrane proteins, ATP and reactive oxygen species (ROS) are also involved in the regulation of cellular responses.^[^ ^83^, ^84^ ^]^

#### 2.2.2 Mechanical Signal

**Figure 3:**
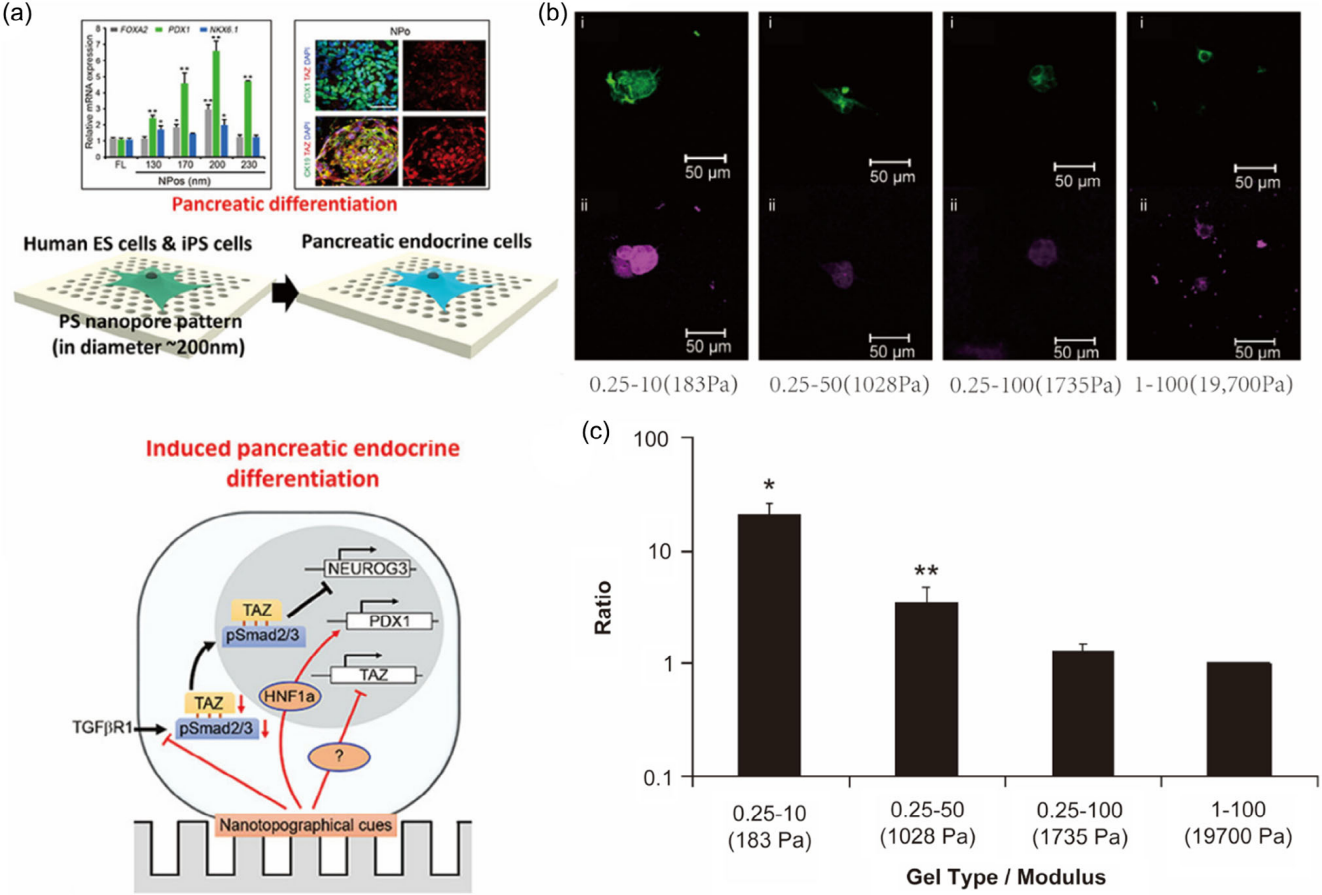
a) Conformation‐mediated cell differentiation. Reproduced with permission.^[^ ^86^ ^]^ Copyright 2016, American Chemical Society. b) Confocal images and c) quantitative real‐time reverse‐transcription PCR (qRT‐PCR) results on the effect of elastic modulus for cell differentiation. Reproduced with permission.^[^ ^102^ ^]^ Copyright 2009, Elsevier.

Attaching cells to a material surface makes the cells more sensitive to mechanical signals from the material surface. These signals, whether originating from various geometries, material stiffness, or mechanical forces generated by material compression and stretching, can be converted into biological signals through interactions between cells and materials, thereby regulating cell physiological activities (**Figure** **3**).^[^ ^85^, ^86^, ^87^, ^88^ ^]^ Currently, a large number of studies have demonstrated the importance of this mechanical signal. A key player in stretch activation in the regulation of stem cell differentiation is the Piezo protein family, which consists of two isoforms: Piezo1 and Piezo2.^[^ ^89^ ^]^ They exhibit exceptional mechanosensitivity and can respond to changes in membrane tension, functioning as mechanosensitive cation channels.^[^ ^90^ ^]^ Piezo2 is involved in mediating gentle touch, while Piezo1 plays a more critical role in mechanical stress‐regulated cell differentiation process.^[^ ^91^ ^]^ Piezo works through Ca^2+^ signaling, which then promotes stem cell proliferation and differentiation through different pathways.^[^ ^92^ ^]^

Cells can respond to the perception of extracellularly adherent nanomorphology to regulate cell behavior by activating intracellular signaling pathways through ligand‐induced extracellular surface receptors.^[^ ^93^, ^94^, ^95^ ^]^ Therefore, the efficiency of cell adhesion plays a key role in this process.^[^ ^96^ ^]^ Microscale adhesion is generated by integrin aggregation, which facilitates extracellular matrix‐to‐cell signaling and is a key transmembrane receptor protein in cellular response to nanomorphology.^[^ ^97^ ^]^ In the extracellular environment, heterodimeric protein receptors composed of *α*‐ and *β*‐integrin subunits bind to peptide ligands such as arginine, glycine, or aspartic acid sequences. Inside the cell, the regulation of cell behavior is based on the cytoskeleton and different signaling pathways.^[^ ^98^, ^99^, ^100^ ^]^ Integrins are sensitive to matrix stiffness and sense changes in the extracellular environment through cell adhesion. Changes in integrin conformation can modulate the cytoskeleton, thereby affecting cell morphology and mechanical properties.^[^ ^101^ ^]^ Different body tissues exhibit distinct stiffness levels, such as neural tissue (≈1 kPa) and bone tissue (≈30 kPa). By mimicking the stiffness of different tissues, cells can be induced to differentiate into specific lineages. For instance, neural stem cells struggle to survive both extremely soft (<0.1 kPa) and very stiff (>100 kPa) stiffnesses. Neural stem cells tend to differentiate into neuronal cells at stiffnesses comparable to brain tissue, while slightly stiffer materials (7–10 kPa) can induce cell differentiation into astrocytes.^[^ ^102^, ^103^ ^]^

## 3 Piezoelectric Material‐Mediated Cell Differentiation

### 3.1 Cell Differentiation Under Piezoelectric Stimulation

Piezoelectric materials have been proven to be effective mediators of biological stimulation, successfully inducing the differentiation of various cell types, including PC12 cells,^[^ ^81^ ^]^ adipose tissue‐derived stem cells,^[^ ^104^, ^105^ ^]^ and human induced pluripotent stem cells.^[^ ^106^, ^107^ ^]^ Commonly used piezoelectric materials can be categorized to organic and inorganic materials. This section aims to elucidate the role of these materials in cell differentiation (**Table** **1**).

**Table 1:** Common piezoelectric materials for cell differentiation

Cell differentiation type	Piezoelectric material	Electrical property	Primary cells	Ref.
Neuron‐like cells	a‐PVDF	*d*_33_ ≈ 24 pC·N^−1^	rBMSCs	[135]
	PVDF	–	rBMSCs	[117]
	PVDF	–	rBMSCs	[69]
Neurons	FeOOH/PVDF	*d*_33_ ≈ 27.2 pC·N^−1^	rBMSCs	[116]
	PVDF	*d*_33_ ≈ −32 pC·N^−1^	hNPCs	[180]
	P(VDF–TrFE)/BaTiO_3_	*d*_31_ ≈ 53.5 pm V^−1^	SH‐SY5Y	[131]
		*g*_31_ ≈ 0.24 mV N^−1^		
Nerve cells	PLA/KNN@PDA	*d*_33_ ≈ 20 pC·N^−1^	Neural stem cells	[153]
	PLLA–PDA	–	Neural stem cells	[181]
	SP@Fe_3_O_4_@BaTiO_3_	–	Neural stem cells	[148]
Osteoblasts	AESO‐10ATP	*d*_33_ ≈ 0.9 pC·N^−1^	BMSCs	[182]
	PVDF‐PPy	–	BMSCs	[183]
	KNN	220.2 pm V^−1^	BMSCs	[129]
	2Mn5Ca‐BT, 2Mn10Ca‐BT	*d*_33_ ≈ 3.71 pC·N^−1^, *d*_33_ ≈ 3.65 pC·N^−1^	BMSCs	[133]
	CS/PHB@ZnO	–	BMSCs	[66]
	TiO_2_@PVDF	*d*_33_ ≈ 20.81 pm V^−1^	rBMSCs	[119]
	PVFT‐BGM	4 < *d*_33_ < 6 pC·N^−1^	mBMSCs	[167]
	SDBTO‐1	*d*_33_ ≈ 13.95 pm V^−1^	hBMSCs	[132]
	Nylon‐11 nanoparticles	–	DPSCs	[184]
	BaTiO_3_/TC4	*d*_33_ ≈ 0.42 pC·N^−1^	MC3T3‐E1	[185]
	WH	–	MC3T3‐E1	[186]
	PVDF/pBT‐4Ag	*d*_33_ ≈ 8.2 pC·N^−1^	MG‐63 cells	[130]
Neurogenic, angiogenic, and osteogenic differentiation	PWH	–	BMSCs	[164]
Odontogenic differentiation	PKNN	–	DPSCs	[40]
Chondrocytes or osteocytes	PLLA/rGO/PDA	–	ATDC5	[122]
Myogenic cells	TSPS	–	hUCBMSCs	[172]

#### 3.1.1 Organic Piezoelectric Materials

PVDF is a material known for its excellent piezoelectric properties, good biocompatibility, thermal stability, and ability to enhance cell adhesion.^[^ ^108^, ^109^, ^110^, ^111^ ^]^ Since its discovery by researchers in 1969,^[^ ^112^ ^]^ PVDF and its composite materials have been extensively studied and have made significant contributions to the biomedical field.^[^ ^113^, ^114^, ^115^ ^]^

**Figure 4:**
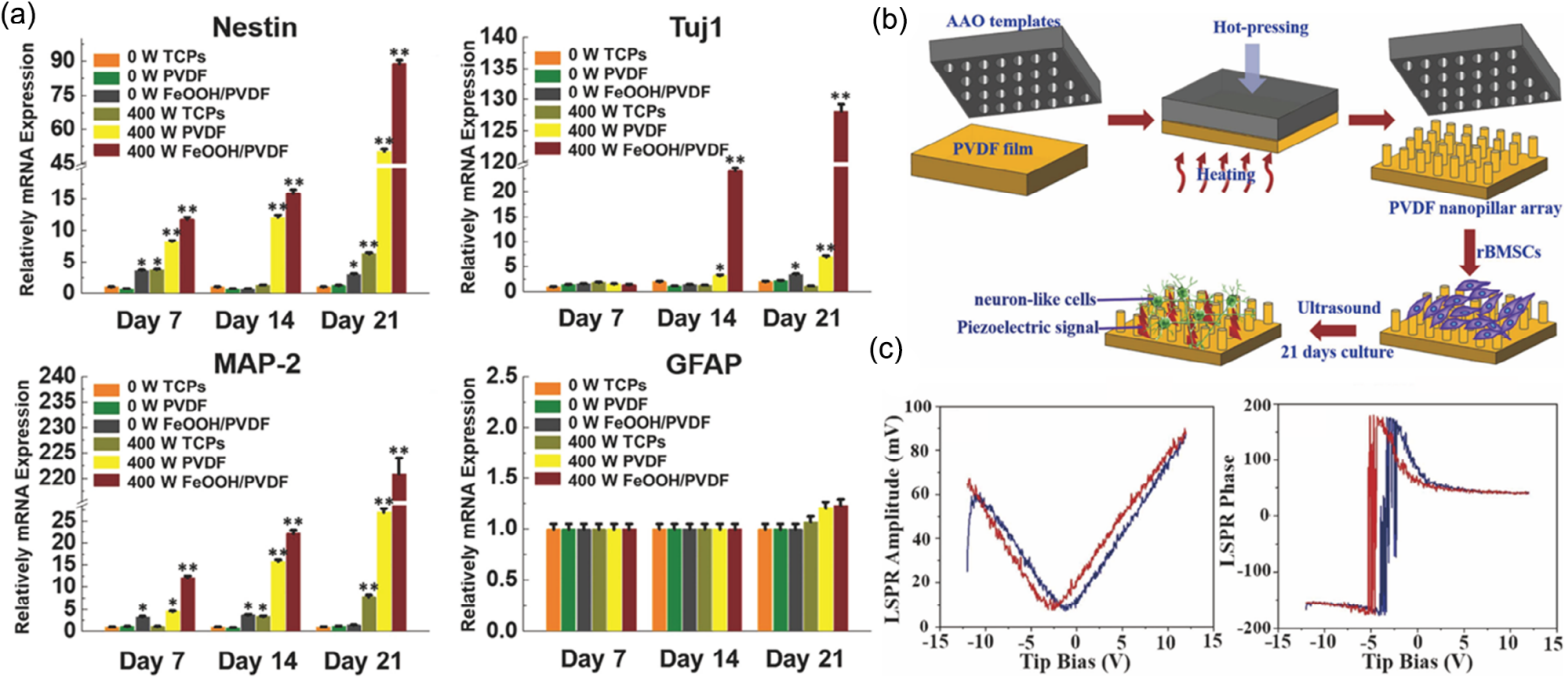
a) Differentiation of rBMSCs on PVDF membranes and PVDF/FeOOH hybrid membranes. Reproduced with permission.^[^ ^116^ ^]^ Copyright 2021, Elsevier. b) Schematic diagram of the preparation of PVDF nanopillars and ultrasound stimulation of cell differentiation and c) amplitude and phase diagrams of the PVDF nanopillars. Reproduced with permission.^[^ ^117^ ^]^ Copyright 2021, Elsevier.

Liu's research group prepared FeOOH/PVDF hybrid membranes by assembling a layer of FeOOH nanorods on a PVDF membrane. Subsequently, electrical stimulation driven by ultrasound irradiation (400 W) was used in synergy with iron ions to regulate the differentiation of rat bone marrow mesenchymal stem cells (rBMSCs) into neurons (**Figure** **4**a).^[^ ^116^ ^]^ In addition, the team took advantage of the easy deformation and point discharge characteristics of nanopillars to create a PVDF pillar array through a template hot pressing method. Compared with PVDF films, PVDF columns are more conducive to generating electrical signals. rBMSCs differentiation into neuron‐like cells was effectively induced using ultrasound stimulation alone without the need for any biological or chemical differentiation factors (Figure 4b,c).^[^ ^117^ ^]^ PVDF is often copolymerized with TrFE to form copolymer P(VDF–TrFE), with the aim of maximizing its crystallinity. It was observed that 3D piezoelectric fiber scaffolds made from P(VDF–TrFE) could effectively stimulate chondrogenic differentiation of MSCs.^[^ ^118^ ^]^ Furthermore, composite PVDF piezoelectric materials constructed by doping nanoparticles often exhibit excellent piezoelectric properties. Titanium dioxide (TiO_2_) has excellent structural stability, surface hydrophilicity, biocompatibility, and antimicrobial properties. The embedded TiO_2_ nanoparticles exert a stretching effect on the PVDF chain and increase the content of *β*‐phase in PVDF. This enables the composite piezoelectric fiber membrane to have excellent piezoelectric properties, which can regulate the ion distribution inside and outside the cells and induce osteogenic differentiation of rBMSCs.^[^ ^119^ ^]^ Additionally, when graphene oxide (GO) or carbon nanotubes (CNTs) are added to PVDF alone, GO increases the β‐phase content through the interactions with the fluorine‐based (–CF2–) segments of PVDF, while the hydrogen atoms on the surface of CNT form hydrogen bonds more readily with the fluorine atoms within PVDF molecules, facilitating the *α* to *β* phase transition. Both piezoelectric materials exhibit improved mechanical properties and piezoelectric effects.^[^ ^120^ ^]^

In addition to PVDF, poly(l‐lactic acid) (PLLA) is another commonly used organic piezoelectric material. Compared with PVDF, PLLA has better biodegradability, and its piezoelectric properties can be effectively enhanced through mechanical stretching.^[^ ^121^ ^]^ Incorporating highly conductive reduced GO into PLLA improves piezoelectric strength and fiber durability, allowing for better control of cell differentiation.^[^ ^122^ ^]^ Other organic piezoelectric materials, such as poly‐*β*‐hydroxybutyrate (PHB), have also been fabricated into scaffolds for tissue engineering applications.^[^ ^123^, ^124^ ^]^ These polymers often have excellent flexibility compared to inorganic piezoelectric materials. However, their shortcomings cannot be ignored. Taking PVDF as an example, its manufacturing process is complex and its production cost is high, which are aspects that need to be considered in practical applications. Furthermore, their geometries are often limited to planar or fibrous forms, which limits their development.

#### 3.1.2 Inorganic Piezoelectric Materials

Most inorganic piezoelectric materials usually exhibit high piezoelectric properties, such as BaTiO_3_,^[^ ^64^, ^125^ ^]^ ZnO,^[^ ^66^, ^126^, ^127^, ^128^ ^]^ and KNN.^[^ ^129^ ^]^ However, they often face challenges in practical applications due to issues such as low toughness and poor deformation resistance.^[^ ^21^ ^]^ Therefore, they are often combined with other materials to enhance overall piezoelectric and mechanical performance. For example, doping BaTiO_3_ into PVDF and introducing silver nanoparticles makes the scaffold flexible while possessing the piezoelectric constant of BaTiO_3_.^[^ ^130^ ^]^ The composite film made of P(VDF–TrFE) and BaTiO_3_ has an increase in the percentage of *β* phase due to the addition of BaTiO_3_ nanoparticles, which significantly improves the piezoelectric properties of the film. The Ca^2+^ transient was then excited by ultrasound stimulation to enhance cell differentiation.^[^ ^131^ ^]^ Moreover, the piezoelectric properties of BaTiO_3_ can be enhanced through doping strategies. Doping sulfur into BaTiO_3_ nanoparticles increases local crystal asymmetry, resulting in a nearly threefold increase in its *d*_33_ relative to pure BaTiO_3_. This confers excellent antimicrobial properties and promotes osteogenic differentiation of stem cells.^[^ ^132^ ^]^ Calcium and manganese (Mn) are intrinsic elements in bone. By doping BaTiO_3_ with an appropriate amount of Ca^2+^/Mn^4+^, excellent osteogenic differentiation induction ability of BMSCs can be demonstrated.^[^ ^133^ ^]^ These works also demonstrate the advantages of piezoelectric materials in inducing cell differentiation by providing physical signals and growth scaffolds.

### 3.2 Cell Differentiation Under Mechanical–Electrical Stimulation

**Figure 5:**
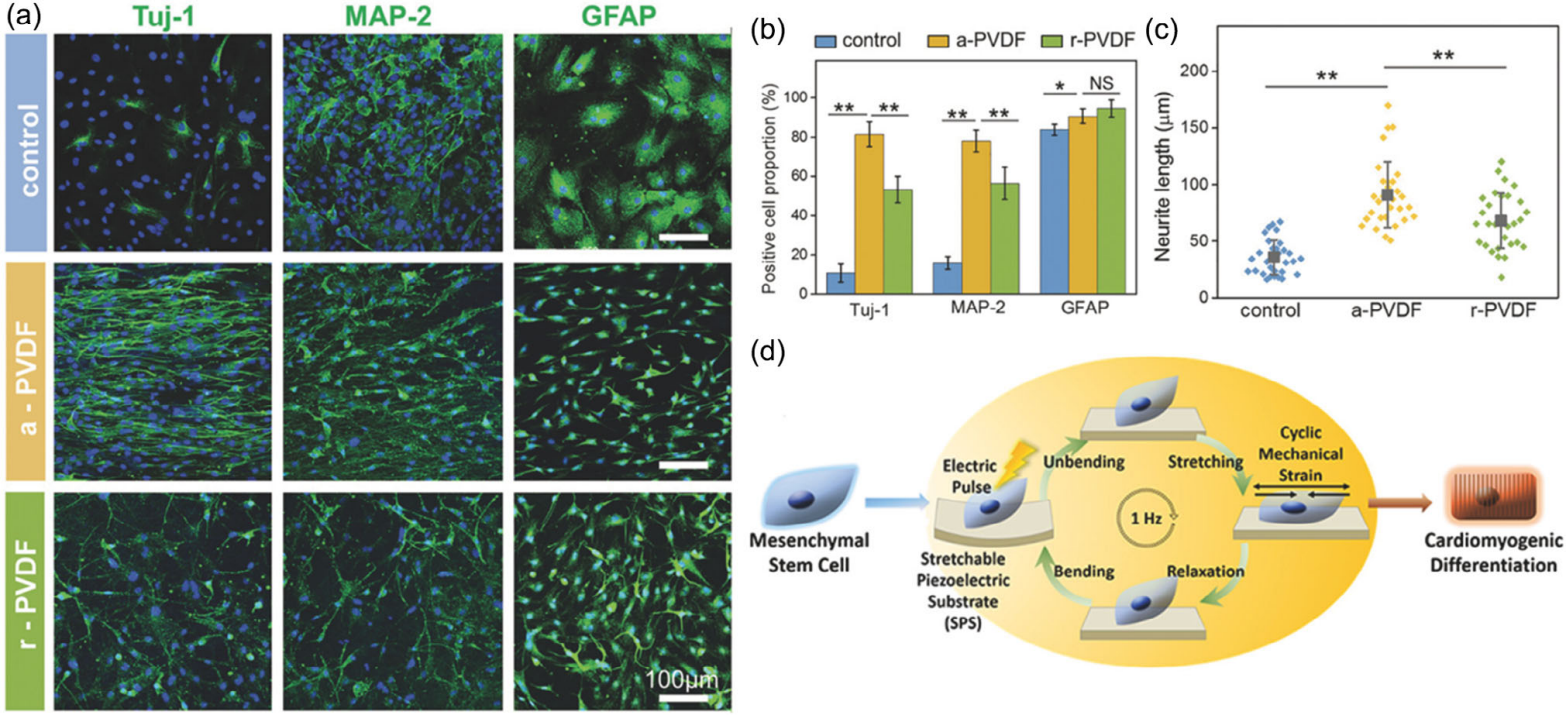
a) Immunofluorescence staining, b) percentage of positive cells, and c) neurite length analysis of rBMSCs differentiation on PVDF nanofibers. Reproduced with permission.^[^ ^135^ ^]^ Copyright 2021, WILEY‐VCH. d) Electrical impulses and mechanical strains generated by bending and cyclic stretching stimulate myocardial differentiation in MSCs. Reproduced with permission.^[^ ^136^ ^]^ Copyright 2017, American Chemical Society.

The mutual contact between cell and piezoelectric material makes cells inevitably affected by mechanical signals on the surface of the material while receiving electrical stimulation. Different material surfaces can exhibit different piezoelectric properties. PVDF nanostripe array structures with two different ridges, grooves, and heights (200 and 500 nm, respectively) exhibit different local piezoelectric potentials when subjected to cellular motion and traction.^[^ ^69^ ^]^ Compared to PVDF‐500, PVDF‐200 has higher crystallinity and piezoelectricity, resulting in the differentiation of a greater number of Tuj‐1‐positive cells. Liu's group constructed electroactive BaTiO_3_/PLLA scaffolds for culturing BMSCs with aligned and randomly oriented fibers.^[^ ^134^ ^]^ In response to electrical stimulation, aligned composite fiber scaffolds were observed to increase cell elongation, which was detrimental to osteogenic differentiation. In contrast, randomly oriented scaffolds had a positive effect on osteogenic differentiation of BMSCs. Similarly, aligned PVDF (a‐PVDF) and randomly aligned PVDF (r‐PVDF) with elastic moduli of 73.1 and 40.5 MPa were prepared by electrospinning. Compared with r‐PVDF, a‐PVDF exhibits superior piezoelectric properties (**Figure** **5**a–c).^[^ ^135^ ^]^ When rBMSCs were cultured on a‐PVDF, the cells expressed more Tju‐1 and MAP‐2 positivity and exhibited longer neurite extension lengths. Mechanical forces also have an influence on cell differentiation. Myoung's team developed a stretchable piezoelectric substrate to stimulate the pulsed electromechanical signals of the cardiac microenvironment in vivo (Figure 5d).^[^ ^136^ ^]^ Mechanical stress induced by cyclic stretch combined with electrical pulses maximizes the in vitro cardiac differentiation of human mesenchymal stem cells.

## 4 Application of Piezoelectric Materials to Stimulate Cell Differentiation

In recent years, an increasing number of studies have involved the induction of cell differentiation through physical signals. The utilization of biocompatible piezoelectric materials, coupled with reliance on external stimuli to induce cell differentiation, has proven to be a very promising therapeutic strategy to promote repair of tissue damage.

### 4.1 Nervous System

Neurological injury poses a huge global challenge due to its complex neural network structure, nonregenerative nature of neurons, limited self‐repair ability, and severe secondary complications.^[^ ^137^, ^138^ ^]^ Neuronal transplantation causes needle trauma, which induces an inflammatory response in the host. A recent study showed that adverse effects during transplantation can be reduced by cotransplantation of regulatory T cells and human‐induced pluripotent stem cell‐derived midbrain dopaminergic neuronal cells.^[^ ^139^ ^]^ However, due to the nondividing nature of neuronal cells, this is still a drop in the bucket for treatment. Glial cells including microglia,^[^ ^140^, ^141^ ^]^ Schwann cells,^[^ ^142^ ^]^ and olfactory sheath cells^[^ ^143^, ^144^ ^]^ play an important role in promoting myelination and axon regeneration, but they are plagued by issues such as shortage of donor cells and ethical issues. The application of electrical pulses to stimulate axonal growth and induce cell differentiation offers potential solutions to these problems. Recent studies have shown that using piezoelectric materials to stimulate cell differentiation produces very promising results in vitro. In addition, these materials can be noninvasively implanted into the body and achieve satisfactory therapeutic effects through precise remote control, significantly enhancing their clinical applicability.^[^ ^145^ ^]^

#### 4.1.1 Central Nervous System

The central nervous system (CNS) consists of the brain and spinal cord and is the basis of human life activities. Due to the nonregenerable nature of neurons, neurodegenerative diseases and physical injuries often lead to irreversible damage and loss of function in the CNS.^[^ ^146^, ^147^ ^]^ Currently, there are many approaches emphasizing neuronal differentiation among the therapeutic strategies for neurodegenerative diseases. For instance, controlled electrical stimulation is used to induce differentiation of neural stem cells into various cell types, including dopaminergic neurons, astrocytes, and oligodendrocytes.^[^ ^148^ ^]^ In a typical example, the combination of nanotopography‐induced mechanical stimulation and piezoelectric material‐triggered electrical stimulation has been effectively promoted BMSC differentiation into *β*‐III tubulin‐positive neurons.^[^ ^69^ ^]^ Moreover, wireless micro–nanorobots fabricated using magnetic nanoparticles and piezoelectric polymers offer a promising avenue. These micro‐ and nanorobots can facilitate cell delivery and induce in situ neural differentiation under ultrasound stimulation. In this innovative approach, magnetic particles are responsible for providing motility, while piezoelectric polymers act as an ultrasound‐responsive electrical stimulation platform.^[^ ^149^ ^]^ Nevertheless, it must be emphasized that these studies are still in the field of in vitro experiments, and their therapeutic effects in the complex environment of the brain require rigorous verification.

Spinal cord injury (SCI) has long been extremely serious condition among CNS diseases. The inherent self‐healing ability of the spinal cord is extremely limited, often resulting in permanent impairment of motor and sensory nerve function.^[^ ^150^, ^151^ ^]^ Polylactic acid (PLA) is a biodegradable biopolymer with piezoelectric properties, although these properties are relatively weak.^[^ ^152^ ^]^ To enhance piezoelectric properties, an effective approach was taken to incorporate an inorganic piezoelectric nanomaterial called KNN into the PLA matrix to construct a piezoelectric PLA/KNN@polydopamine (PLA/KNN@PDA) composite nanofiber scaffold.^[^ ^153^ ^]^ As the KNN@PDA mass ratio (0–50%) increases, the peak output voltage of the nanoscaffold triggered under pressure (0.5 N, 10 Hz) increased sharply, from 0.52 V to 17.9 V. The output current increased from 0.12 to 2.6 μA, and the output efficiency was significantly improved. The piezoelectric coefficient of the PLA/KNN@PDA (50%) composite is comparable to that of the widely used PVDF. Neural stem cells were cultured under neuronal conditions for 7 days and irradiated with ultrasound (100 kPa, 1 MHz) for 20 min every day. PLA/KNN@PDA effectively promoted the neural differentiation of NSCs. After the PLA/KNN@PDA scaffold was implanted into the SCI site, it not only provided a favorable matrix environment for the growth of neural stem cells, but also promoted cell differentiation through external ultrasound stimulation and carefully planned controlled electrical stimulation. The results are revealing, showing that the use of piezoelectric scaffolds in combination with ultrasound‐driven electrical stimulation significantly reduced spinal cord tissue loss following trauma. It also enhanced myelin preservation and inhibited astrocyte responses to injury. Notably, the expression of brain‐derived neurotrophic factor, its specific receptor, tyrosine kinase B (TrkB), and vascular endothelial markers was significantly upregulated. This cascade of events ultimately leads to recovery of motor function and enhanced repair of SCI.

#### 4.1.2 Peripheral Nervous System

The peripheral nervous system (PNS) encompasses virtually all neural structures except the brain and spinal cord, including the regulation of sensory input, motor output, and autonomic functions such as heartbeat and blood pressure.^[^ ^154^ ^]^ Therefore, peripheral nerve injury (PNI) should not be underestimated. It often results in partial loss of sensory and motor functions, bringing survival pressure and economic burden to patients.

**Figure 6:**
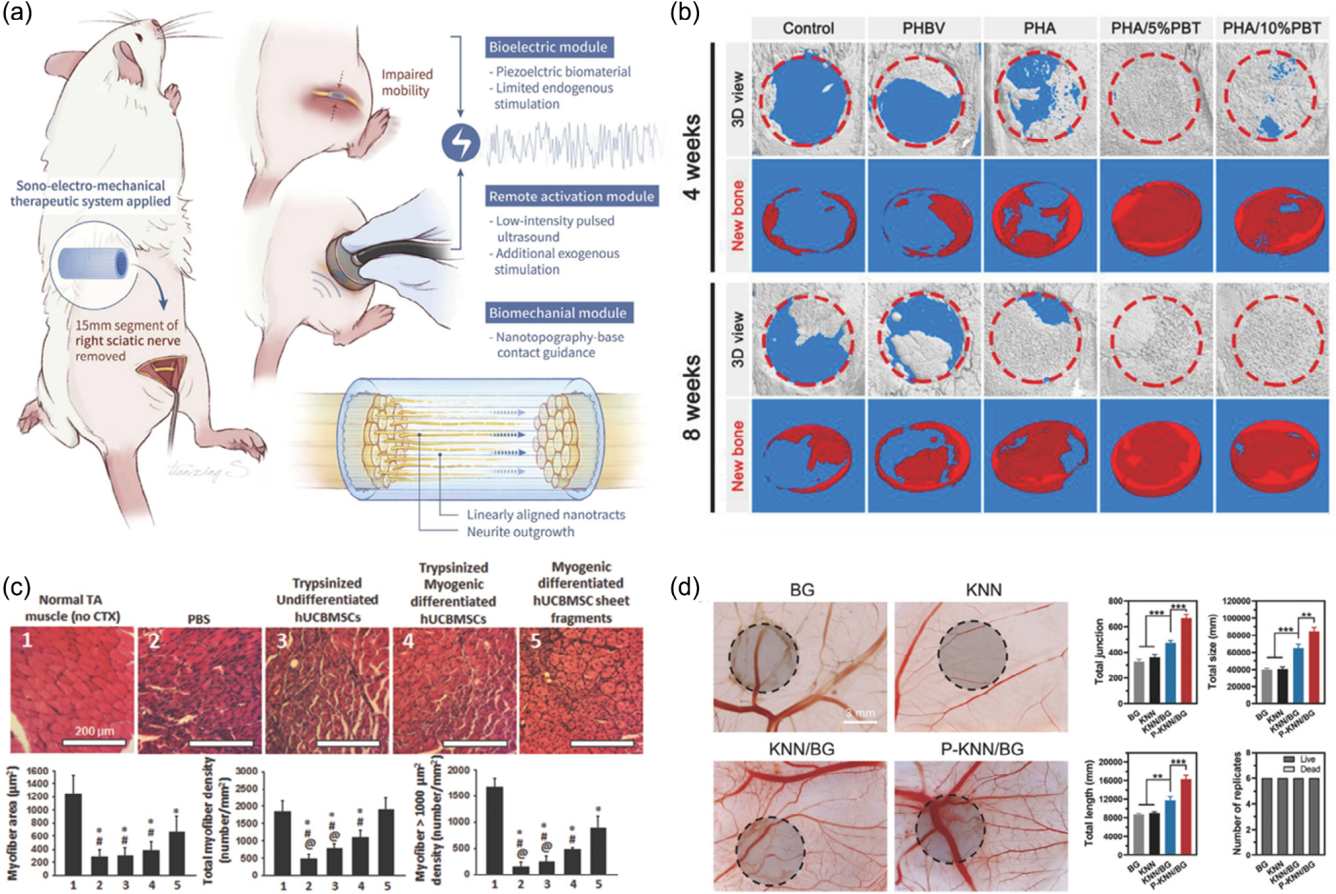
a) Schematic diagram of acoustic electromechanical treatment of PNI. Reproduced with permission.^[^ ^162^ ^]^ Copyright 2023, Elsevier. b) In vivo assessment of bone regeneration 3D micro‐CT images. Reproduced with permission.^[^ ^166^ ^]^ Copyright 2023, American Chemical Society. c) Assessment of skeletal muscle regeneration after 10 days of injection of myofibroblast‐differentiated hUCBMSCs fragments. Reproduced with permission.^[^ ^172^ ^]^ Copyright 2017, WILEY‐VCH. d) In vivo assessment of angiogenesis were, respectively, optical images of CAMs after 5 days of stimulation (gray shaded portions indicate the location where the samples were placed), the total vascular junctions, the total size, the total length, and the survival of chicken embryos. Reproduced with permission.^[^ ^179^ ^]^ Copyright 2023, WILEY‐VCH.

The skin is the largest organ of the human body.^[^ ^155^ ^]^ Skin nerve regeneration is a complex process. Peng's group investigated a self‐powered functional patch composed of a flexible piezoelectric film and a conductive gel that provides electrical stimulation.^[^ ^156^ ^]^ This strategy effectively accelerated nerve regeneration and sensory recovery at the wound site within 23 days. Furthermore, in clinical practice, autologous nerve grafting is considered the gold standard for the treatment of persistent PNI, such as sciatic nerve injuries. However, due to the limitations of autografts, new tissue engineering alternatives need to be developed to address this issue.^[^ ^157^, ^158^, ^159^, ^160^ ^]^ PVDF shows excellent results in sciatic nerve repair. Cao's team combined poly(l‐lactide*‐co*‐ε‐caprolactone) (PLCL) and PVDF to construct a composite piezoelectric film PVDF/PLCL.^[^ ^161^ ^]^ This composite was wrapped in a filipin protein/poly(3,4‐ethylenedioxythiophene) scaffold to enhance its biodegradability and electrical conductivity. In a rat 10 mm sciatic nerve defect model, this approach promoted axonal regeneration, myelination, neurovascularization, and neurological recovery. At 12 weeks, the therapeutic effect reached a level similar to that of autologous nerve transplantation. Moreover, Sun's group combined polycaprolactone (PCL) and PVDF to create linearly aligned piezoelectric nanobundles. In vitro experiments showed that culturing Schwann cells on PCL/PVDF nanobundles and applying low‐intensity pulsed ultrasound (20 min per day) for 5 days effectively promoted Schwann cell proliferation and the expression of neurotrophic factors. And by stimulating PC12 cells, the feasibility of acoustic mechanical electrical therapy to promote the growth and differentiation of nerve cells was verified. Moreover, in vivo application of PCL/PVDF nanobundles demonstrated its effects on motor function recovery and axonal maturation, thereby enhancing its clinical potential (**Figure** **6**a).^[^ ^162^ ^]^

### 4.2 Bone

Bones are primarily composed of mineralized complexes of compounds such as carbonized hydroxyapatite and collagen.^[^ ^163^ ^]^ Their natural composition confers intrinsic piezoelectric properties,^[^ ^164^ ^]^ providing the necessary biophysical factors for cell proliferation and differentiation. Currently, osteogenic differentiation based on piezoelectric materials has been extensively studied. Using appropriate piezoelectric materials to fill bone defects and simulate the endogenous electrical environment of the bone as a generator to promote cell differentiation and enhance bone regeneration is an important aspect of current bone regeneration research.

Nguyen's team prepared biocompatible and biodegradable PLLA piezoelectric films and implanted them into cranial defects in mice.^[^ ^165^ ^]^ They applied external ultrasound to stimulate osteogenic differentiation of adipose‐derived stem cells and BMSCs to achieve bone regeneration. Qi's group constructed a piezoelectric periosteum with biocompatibility, osteogenic activity, and immunomodulatory properties by mixing poly(3‐hydroxybutyrate*‐co*‐3‐hydrovaleric acid), antioxidant polydopamine‐modified hydroxyapatite, and BaTiO_3_.^[^ ^166^ ^]^ After the material is implanted into the rat skull defect area, it has significant ROS scavenging activity, promotes the M1–M2 phenotype transformation of macrophages, and improves the local injury microenvironment. Electrical stimulation enhanced BMSC adhesion, proliferation, and osteogenic differentiation, strengthening new bone vascularization and regeneration capability (Figure 6b). In addition, Chen's team incorporated inorganic minerals calcium and phosphorus into the biocompatible biopiezoelectric scaffold PVDF–TrFE to simulate bone matrix.^[^ ^167^ ^]^ The synthesized scaffold significantly improved the proliferation, adhesion, and osteogenic differentiation of BMSCs. This enhancement is likely attributed to the accumulation of Ca^2+^ that activate the Ca‐sensitive receptor (CaSR). Once CaSR is activated, its intracellular region interacts with many downstream molecules to regulate different signaling pathways in cellular activity. This kind of transplantation is beneficial to the formation of periosteal‐like tissue and bone regeneration in the bone defect area.

### 4.3 Muscle Tissue

Muscle tissue is mainly classified into skeletal muscle, cardiac muscle, and smooth muscle.^[^ ^168^ ^]^ Skeletal muscle is one of the most abundant tissues in the human body, composed of aligned cells and extracellular matrix, and accounts for approximately 40–50% of body weight.^[^ ^169^ ^]^ When a traumatic injury is sustained, permanent disability may result due to incomplete regeneration of skeletal muscle, particularly in cases of severe injury or surgical removal of the muscle or muscle unit. Therefore, the use of certain biophysical stimuli to promote cell proliferation and differentiation has proven to be a very effective approach.^[^ ^64^, ^170^, ^171^ ^]^ Lee et al. combined electrical and mechanical stimulation to treat sarcopenia (Figure 6c).^[^ ^172^ ^]^ Biaxially stretched ZnO nanorods provide the piezoelectric pulses, polydimethylsiloxane provides the mechanical strain, and thermosensitive poly(*N*‐isopropylacrylamide) is used as the surface coating. Adherent human umbilical cord blood mesenchymal stem cells (hUCBMSCs) were provided mechanical and electrical stimulation by repeated bending and stretching at a frequency of 0.3 Hz for 10 days. These cells differentiated into myogenic cells and were injected into mice with cardiotoxin‐induced skeletal muscle injury, which enhanced muscle regeneration. In addition, Ribeiro's team introduced a new approach to apply magnetoelectric coupling to skeletal muscle repair.^[^ ^173^ ^]^ They utilized a composite material composed of piezoelectric P(VDF–TrFE) and magnetostrictive nanoparticles–cobalt ferrite nanoparticles (CoFe_2_O_4_). This composite was designed to take advantage of the magnetoelectric effect, where the magnetostrictive CoFe_2_O_4_ deformed under the influence of an external magnetic field, and the piezoelectric properties of P(VDF–TrFE) were triggered by the external magnetic field, resulting in an electric response.

### 4.4 Blood Vessel

Blood vessels play a crucial role in transporting nutrients and oxygen and removing metabolic waste products, making them essential for tissue repair and regeneration.^[^ ^174^ ^]^ Their involvement is evident in various regenerative processes, such as bone tissue regeneration,^[^ ^63^, ^164^, ^175^ ^]^ nerve tissue regeneration,^[^ ^176^, ^177^ ^]^ and skin regeneration.^[^ ^178^ ^]^ Zhai's team observed the apparent ability of piezoelectric composites to promote angiogenesis through experiments on chorioallantoic membrane of chicken embryos (Figure 6d).^[^ ^179^ ^]^ They synthesized polarized KNN (PKNN) using piezoelectric bioactive glass (BG) composites (PKNN/BG). PKNN/BG had a higher surface potential (332.04 ± 12.85 mV) than BG and KNN/BG as measured by scanning Kelvin probe force microscopy and was able to provide excellent radiation stimulation to seed human umbilical vein endothelial cells onto its surface. The combined effect of electrical stimulation and reactive ions enhanced endothelial cell adhesion, migration, and differentiation by activating the eNOS/NO signaling pathway to upregulate Ca^2+^ levels and increase the expression of VEGF, b‐FGF, and TGF‐β.

## 5 Discussion

It is well known that piezoelectric nanomaterials have great potential in regulating cell differentiation to promote tissue regeneration. In recent years, with the deepening of research on the role of physical signals (electrical, mechanical, etc.) in stem cell differentiation, piezoelectric nanomaterials have received great attention as functional medical materials that can generate electrical signals. They offer the advantages of easy application, stable performance, and high cost‐effectiveness. These materials can be directly implanted into the body, where electrical signals synergize with surface mechanical signals to enhance cell adhesion, proliferation, and differentiation. Moreover, their stable energy supply has found wide applications in biosensing, especially in monitoring vital signs and disease diagnosis. Tissue regenerative medicine based on piezoelectric nanomaterials represents a unique branch of tissue engineering. It relies on the interdisciplinary convergence of multiple fields such as materials science, molecular biology, biophysics, chemistry, regenerative medicine, and so on, making it a complex undertaking. As an emerging and highly interdisciplinary subject, our current understanding is only on the surface, and this field is likely to encounter numerous challenges in the future.

Biosafety is a critical concern in almost all biomedical disciplines. Although many biocompatible piezoelectric nanomaterials have been explored, long‐term implantation of these materials requires rigorous safety assessment. Comprehensive validation is needed to assess the cytotoxicity, potential inflammatory responses, and in vivo metabolic capabilities of nanomaterials. On the other hand, the cavitation effect generated by ultrasound is known to cause a sharp increase in ROS, leading to significant cellular damage and being detrimental to tissue regeneration. This issue can be mitigated by loading materials with ROS scavenging capabilities to reduce their adverse effects. Alternatively, constructing biomaterials that are conducive to cell growth could help mitigate the impact of the microenvironment on cell growth after injury.

PFM is widely recognized for evaluating the piezoelectric properties of nanomaterials. However, when measuring piezoelectric nanomaterials that are very small in size or have extremely smooth surfaces, the piezoelectric probe may not land on them accurately, which may lead to measurement errors or difficulty in replicating the results. Additionally, when comparing the piezoelectric properties of multiple nanomaterials with relatively small differences (less than one order of magnitude), it can be challenging to accurately assess which nanomaterial exhibits superior piezoelectric properties. Better‐performing nanomaterials require less mechanical stimulation to produce the same electrical signal. This can effectively reduce the adverse effects of external mechanical forces on cells and tissues, which is of great significance for tissue regeneration. Therefore, methods to evaluate piezoelectric properties still need to be further explored.

In recent years, research on cell differentiation induced by physical signals has become more in‐depth. Light, electricity, heat, sound, magnetism, and mechanical forces have all been found to be used to regulate cell fate. Although researchers have identified various regulatory pathways, including Ca^2+^ signaling, we are still limited by the complex regulatory networks within cells, which require further investigation. Additionally, piezoelectric stimulation affects cells in vivo differently than in vitro conditions. This requires consideration of how the in vivo microenvironment after injury interferes with cell differentiation and how electrical stimulation affects tissue repair. Addressing these challenges is critical to advancing the field of piezoelectric nanomaterials for tissue regeneration.

Piezoelectric nanomaterials, when integrated with mechanical and electrical signals, can jointly induce cell differentiation for optimal outcomes. They can also be combined with other nanomaterials, showcasing great potential. For instance, the introduction of silver nanoparticles enhances the antibacterial properties of the material, and the use of magnetic nanoparticles promotes in situ neuronal differentiation, significantly improving the therapeutic effect. Piezoelectric nanomaterials do not require additional wires and external power sources like traditional electrical stimulation therapies, and direct injection into the injured area through a needle can truly achieve localized, noninvasive treatment.

In summary, although piezoelectric nanomaterials have made significant contributions in various fields, their research in the field of tissue regeneration is still in its infancy. With the rapid advancement of new technologies and the continuous deepening of interdisciplinary research, future cell therapy based on piezoelectric materials may truly solve the problem of cell shortage and pave the way for the treatment of tissue damage.

## Conflict of Interest

The authors declare no conflict of interest.

## References

[1] L. Meng , N. Ren , M. Dong , S. Zhang , A. Wang , Z. Zhuang , J. Wang , C. Sun , H. Liu , Adv. Funct. Mater. 2023, 10.1002/adfm.202309974.

[2] W. Xue , W. Shi , Y. Kong , M. Kuss , B. Duan , Bioact. Mater. 2021, 6, 4141. 33997498 10.1016/j.bioactmat.2021.04.019 PMC8099454

[3] X. Wang , K. Xu , L. Mu , X. Zhang , G. Huang , M. Xing , Z. Li , J. Wu , Adv. Healthcare Mater. 2023, 12, 2203400. 10.1002/adhm.202203400 37462927

[4] Q. Yu , J. Chen , W. Deng , X. Cao , Y. Wang , J. Zhou , W. Xu , P. Du , Q. Wang , J. Yu , X. Xu , J. Nanobiotechnology 2017, 15, 82. 29137640 10.1186/s12951-017-0317-y PMC5686901

[5] R. Langer , J. P. Vacanti , Science 1993, 260, 920. 8493529 10.1126/science.8493529

[6] R. D. Pedde , B. Mirani , A. Navaei , T. Styan , S. Wong , M. Mehrali , A. Thakur , N. K. Mohtaram , A. Bayati , A. Dolatshahi‐Pirouz , M. Nikkhah , S. M. Willerth , M. Akbari , Adv. Mater. 2017, 29, 1606061. 10.1002/adma.201606061 28370405

[7] F. K. Lewns , O. Tsigkou , L. R. Cox , R. D. Wildman , L. M. Grover , G. Poologasundarampillai , Adv. Mater. 2023, 35, 2302670. 10.1002/adma.202301670 PMC11478930 37087739

[8] D. N. Tavakol , S. Fleischer , G. Vunjak‐Novakovic , Cell Stem Cell 2021, 28, 993. 34087161 10.1016/j.stem.2021.05.008 PMC8186820

[9] L. Tullie , B. C. Jones , P. De Coppi , V. S. W. Li , Nat. Rev. Gastro. Hepat. 2022, 19, 417. 10.1038/s41575-022-00586-x 35241800

[10] L. G. Griffith , G. Naughton , Science 2002, 295, 1009. 11834815 10.1126/science.1069210

[11] X.‐R. Ong , A. X. Chen , N. Li , Y. Y. Yang , H.‐K. Luo , Small Sci. 2023, 3, 2200076. 10.1002/smsc.202200076 PMC11935994 40213493

[12] F. Ye , Z. Yan , H. Zhang , H. Chang , P. Neuzil , Trac‐trend. Anal. Chem. 2020, 126, 115858.

[13] B. Lo , L. Parham , Endocr. Rev. 2009, 30, 204. 19366754 10.1210/er.2008-0031 PMC2726839

[14] Y. Kong , J. Duan , F. Liu , L. Han , G. Li , C. Sun , Y. Sang , S. Wang , F. Yi , H. Liu , Chem. Soc. Rev. 2021, 50, 12828. 34661592 10.1039/d1cs00572c

[15] G. Zhao , H. Zhou , G. Jin , B. Jin , S. Geng , Z. Luo , Z. Ge , F. Xu , Prog. Polym. Sci. 2022, 131, 101573.

[16] S. R. Das , M. Uz , S. Ding , M. T. Lentner , J. A. Hondred , A. A. Cargill , D. S. Sakaguchi , S. Mallapragada , J. C. Claussen , Adv. Healthcare Mater. 2017, 6, 1601087. 10.1002/adhm.201601087 28218474

[17] A. B. Ribera , N. C. Spitzer , Neuron 1989, 2, 1055. 2483107 10.1016/0896-6273(89)90229-8

[18] K. H. Arulkandarajah , G. Osterstock , A. Lafont , H. Le Corronc , N. Escalas , S. Corsini , B. Le Bras , L. Chenane , J. Boeri , A. Czarnecki , C. Mouffle , E. Bullier , E. Hong , C. Soula , P. Legendre , J.‐M. Mangin , Curr. Biol. 2021, 31, 4584. 34478646 10.1016/j.cub.2021.08.019

[19] R. Balint , N. J. Cassidy , S. H. Cartmell , Tissue Eng. Part B Rev. 2013, 19, 48. 22873689 10.1089/ten.TEB.2012.0183

[20] J. Li , Y. Feng , W. Chen , S. Zhang , J. Ma , S. Chen , S. Liu , C. Cao , Y. Zhang , Prog. Mater. Sci. 2023, 132, 101045.

[21] X. Zhang , T. Wang , Z. Zhang , H. Liu , L. Li , A. Wang , J. Ouyang , T. Xie , L. Zhang , J. Xue , W. Tao , Mater. Today 2023, 68, 177.

[22] Z. Liu , X. Wan , Z. L. Wang , L. Li , Adv. Mater. 2021, 33, 2007429. 10.1002/adma.202007429 34117803

[23] Y. Wang , K.‐K. Liu , W.‐B. Zhao , J.‐L. Sun , X.‐X. Chen , L.‐L. Zhang , Q. Cao , R. Zhou , L. Dong , C.‐X. Shan , Nano Today 2023, 48, 101737.

[24] A. S. Verma , A. Singh , D. Kumar , A. K. Dubey , ACS Biomater. Sci. Eng. 2020, 6, 3055. 33463258 10.1021/acsbiomaterials.0c00091

[25] A. Wu , L. Jiang , C. Xia , Q. Xu , B. Zhou , Z. Jin , Q. He , J. Guo , Adv. Sci. 2023, 10, 2303016. 10.1002/advs.202303016 PMC10558630 37587791

[26] F. Lv , J. Lin , Z. Zhou , Z. Hong , Y. Wu , Z. Ren , Q. Zhang , S. Dong , J. Luo , J. Shi , R. Chen , B. Liu , Y. Su , Y. Huang , Nano Energy 2022, 100, 107507.

[27] G. Yang , Y. Tang , T. Lin , T. Zhong , Y. Fan , Y. Zhang , L. Xing , X. Xue , Y. Zhan , Nano Energy 2022, 93, 106817.

[28] C. A. Bassett , R. O. Becker , Science 1962, 137, 1063. 13865637 10.1126/science.137.3535.1063

[29] H. Athenstaedt , H. Claussen , D. Schaper , Science 1982, 216, 1018. 6177041 10.1126/science.6177041

[30] D. Vasilescu , R. Cornillon , G. Mallet , Nature 1970, 225, 635. 5413369 10.1038/225635a0

[31] W. Fang , H. Ping , X. Li , X. Liu , F. Wan , B. Tu , H. Xie , P. O’Reilly , H. Wang , W. Wang , Z. Fu , Adv. Funct. Mater. 2021, 31, 2105806.

[32] F. R. Fan , W. Tang , Z. L. Wang , Adv. Mater. 2016, 28, 4283. 26748684 10.1002/adma.201504299

[33] S.‐G. Kwon , B.‐H. Park , K. Choi , E.‐S. Choi , S. Nam , J.‐W. Kim , J.‐H. Kim , J. Eur. Ceram. Soc. 2006, 26, 1401.

[34] G. H. Kwei , A. C. Lawson , S. J. L. Billinge , S. W. Cheong , J. Phys. Chem. 1993, 97, 2368.

[35] P. Chen , P. Wu , X. Wan , Q. Wang , C. Xu , M. Yang , J. Feng , B. Hu , Z. Luo , Nano Energy 2021, 86, 106123.

[36] Z. Ge , Y. Qiao , W. Zhu , Y. Xu , Q. Fang , D. Wang , Y. Tang , R. Zhao , X. Deng , W. Lin , G. Wang , Y. Xiang , X. Hu , Nano Energy 2023, 115, 108751.

[37] W. M. Grill , Expert Rev. Med. Devices 2005, 2, 409. 16293080 10.1586/17434440.2.4.409

[38] G. Xia , B. Song , J. Fang , Research 2022, 2022, 9896274. 36061820 10.34133/2022/9896274 PMC9394050

[39] C. Li , C. Xiao , L. Zhan , Z. Zhang , J. Xing , J. Zhai , Z. Zhou , G. Tan , J. Piao , Y. Zhou , S. Qi , Z. Wang , P. Yu , C. Ning , Bioact. Mater. 2022, 18, 399. 35415302 10.1016/j.bioactmat.2022.03.027 PMC8965767

[40] J. Zheng , J. Zuo , C. Xiao , Q. Guo , X. Fu , C. Ning , P. Yu , J. Mater. Sci. Technol. 2024, 168, 24.

[41] J. Rödel , J.‐F. Li , MRS Bull. 2018, 43, 576.

[42] M. T. Chorsi , E. J. Curry , H. T. Chorsi , R. Das , J. Baroody , P. K. Purohit , H. Ilies , T. D. Nguyen , Adv. Mater. 2019, 31, 1802084. 10.1002/adma.201802084 30294947

[43] B. Jiang , J. Iocozzia , L. Zhao , H. Zhang , Y.‐W. Harn , Y. Chen , Z. Lin , Chem. Soc. Rev. 2019, 48, 1194. 30663742 10.1039/c8cs00583d

[44] A. Sood , M. Desseigne , A. Dev , L. Maurizi , A. Kumar , N. Millot , S. S. Han , Small 2023, 19, 2206401. 10.1002/smll.202206401 36585372

[45] L. Kou , W. Guo , C. Li , in 2008 Symposium on Piezoelectricity, Acoustic Waves, and Device Applications, IEEE, Nanjing, China 2008, pp. 354–359.

[46] A. Cafarelli , A. Marino , L. Vannozzi , J. Puigmartí‐Luis , S. Pané , G. Ciofani , L. Ricotti , ACS Nano 2021, 15, 11066. 34251189 10.1021/acsnano.1c03087 PMC8397402

[47] K. C. Satyanarayana , K. Bolton , Polymer 2012, 53, 2927.

[48] Q. Li , Q. Wang , Macromol. Chem. Phys. 2016, 217, 1228.

[49] C. Wan , C. R. Bowen , J. Mater. Chem. A 2017, 5, 3091.

[50] J. Zhang , X. Wang , X. Chen , X. Xia , G. J. Weng , Energy Convers. Manag. 2022, 269, 116121.

[51] D. Kim , S. A. Han , J. H. Kim , J.‐H. Lee , S.‐W. Kim , S.‐W. Lee , Adv. Mater. 2020, 32, 1906989. 10.1002/adma.201906989 32103565

[52] R. Wang , J. Sui , X. Wang , ACS Nano 2022, 16, 17708. 36354375 10.1021/acsnano.2c08164 PMC10040090

[53] A. Dawson , D. R. Allan , S. A. Belmonte , S. J. Clark , W. I. F. David , P. A. McGregor , S. Parsons , C. R. Pulham , L. Sawyer , Cryst. Growth Des. 2005, 5, 1415.

[54] A. Heredia , V. Meunier , I. K. Bdikin , J. Gracio , N. Balke , S. Jesse , A. Tselev , P. K. Agarwal , B. G. Sumpter , S. V. Kalinin , A. L. Kholkin , Adv. Funct. Mater. 2012, 22, 2996.

[55] S. Guerin , A. Stapleton , D. Chovan , R. Mouras , M. Gleeson , C. McKeown , M. R. Noor , C. Silien , F. M. F. Rhen , A. L. Kholkin , N. Liu , T. Soulimane , S. A. M. Tofail , D. Thompson , Nat. Mater. 2018, 17, 180. 29200197 10.1038/nmat5045

[56] Q. Niu , H. Wei , B. S. Hsiao , Y. Zhang , Nano Energy 2022, 96, 107101.

[57] H. K. Ravi , F. Simona , J. Hulliger , M. Cascella , J. Phys. Chem. B 2012, 116, 1901. 22242946 10.1021/jp208436j

[58] V. Sencadas , C. Garvey , S. Mudie , J. J. K. Kirkensgaard , G. Gouadec , S. Hauser , Nano Energy 2019, 66, 104106.

[59] T. Yucel , P. Cebe , D. L. Kaplan , Adv. Funct. Mater. 2011, 21, 779. 23335872 10.1002/adfm.201002077 PMC3546528

[60] M. H. Shamos , L. S. Lavine , Nature 1967, 213, 267. 6030604 10.1038/213267a0

[61] C. R. West , A. E. Bowden , Ann. Biomed. Eng. 2012, 40, 1568. 22314836 10.1007/s10439-011-0504-1

[62] F. Vasquez‐Sancho , A. Abdollahi , D. Damjanovic , G. Catalan , Adv. Mater. 2018, 30, 1705316. 10.1002/adma.201705316 29345377

[63] B. Wang , G. Li , Q. Zhu , W. Liu , W. Ke , W. Hua , Y. Zhou , X. Zeng , X. Sun , Z. Wen , C. Yang , Y. Pan , Small 2022, 18, 2201056. 10.1002/smll.202201056 35652171

[64] C. Paci , F. Iberite , L. Arrico , L. Vannozzi , P. Parlanti , M. Gemmi , L. Ricotti , Biomater. Sci. 2022, 10, 5265. 35913209 10.1039/d1bm01853a

[65] G. G. Genchi , E. Sinibaldi , L. Ceseracciu , M. Labardi , A. Marino , S. Marras , G. De Simoni , V. Mattoli , G. Ciofani , Nanomedicine. 2018, 14, 2421. 28552646 10.1016/j.nano.2017.05.006

[66] Z. Chen , J. Zheng , X. Pei , S. Sun , J. Cai , Y. Liu , Y. Wang , L. Zheng , H. Zhou , Chem. Eng. J. 2023, 470, 144305.

[67] X. Xu , X. Yao , K. Jiang , Y. Zhou , W. Lu , W. Jiang , X. Wang , J. Clean. Prod. 2022, 359, 132070.

[68] C. Wang , W. Sun , Y. Xiang , S. Wu , Y. Zheng , Y. Zhang , J. Shen , L. Yang , C. Liang , X. Liu , Small Sci. 2023, 3, 2300022. 10.1002/smsc.202300022 PMC11935888 40212404

[69] X. Zhang , X. Cui , D. Wang , S. Wang , Z. Liu , G. Zhao , Y. Zhang , Z. Li , Z. L. Wang , L. Li , Adv. Funct. Mater. 2019, 29, 1900372.

[70] P. Güthner , K. Dransfeld , Appl. Phys. Lett. 1992, 61, 1137.

[71] P. Buragohain , H. Lu , C. Richter , T. Schenk , P. Kariuki , S. Glinsek , H. Funakubo , J. Íñiguez , E. Defay , U. Schroeder , A. Gruverman , Adv. Mater. 2022, 34, 2206237. 10.1002/adma.202206237 36210741

[72] N. Balke , P. Maksymovych , S. Jesse , A. Herklotz , A. Tselev , C.‐B. Eom , I. I. Kravchenko , P. Yu , S. V. Kalinin , ACS Nano 2015, 9, 6484. 26035634 10.1021/acsnano.5b02227

[73] Y.‐M. You , W.‐Q. Liao , D. Zhao , H.‐Y. Ye , Y. Zhang , Q. Zhou , X. Niu , J. Wang , P.‐F. Li , D.‐W. Fu , Z. Wang , S. Gao , K. Yang , J.‐M. Liu , J. Li , Y. Yan , R.‐G. Xiong , Science 2017, 357, 306. 28729511 10.1126/science.aai8535

[74] G. Hernandez‐Cuevas , J. R. Leyva Mendoza , P. E. García‐Casillas , C. A. Rodríguez González , J. F. Hernandez‐Paz , G. Herrera‐Pérez , L. Fuentes‐Cobas , S. Díaz de la Torre , O. Raymond‐Herrera , H. Camacho‐Montes , J. Adv. Ceram. 2019, 8, 278.

[75] K. Xu , J. Li , X. Lv , J. Wu , X. Zhang , D. Xiao , J. Zhu , Adv. Mater 2016, 28, 8519. 27441456 10.1002/adma.201601859

[76] K. Kapat , Q. T. H. Shubhra , M. Zhou , S. Leeuwenburgh , Adv. Funct. Mater. 2020, 30, 1909045.

[77] M. Zayzafoon , J. Cell. Biochem. 2006, 97, 56. 16229015 10.1002/jcb.20675

[78] M. R. Cho , H. S. Thatte , R. C. Lee , D. E. Golan , FASEB J. 1996, 10, 1552. 8940302 10.1096/fasebj.10.13.8940302

[79] F. Jia , S. Lin , X. He , J. Zhang , S. Shen , Z. Wang , B. Tang , C. Li , Y. Wu , L. Dong , K. Cheng , W. Weng , ACS Appl. Mater. Interfaces 2019, 11, 22218. 31199127 10.1021/acsami.9b07161

[80] G. Thrivikraman , S. K. Boda , B. Basu , Biomaterials 2018, 150, 60. 29032331 10.1016/j.biomaterials.2017.10.003

[81] M. Hoop , X.‐Z. Chen , A. Ferrari , F. Mushtaq , G. Ghazaryan , T. Tervoort , D. Poulikakos , B. Nelson , S. Pané , Sci. Rep. 2017, 7, 4028. 28642614 10.1038/s41598-017-03992-3 PMC5481323

[82] X. Li , B. C. Heng , Y. Bai , Q. Wang , M. Gao , Y. He , X. Zhang , X. Deng , X. Zhang , Bioact. Mater. 2023, 20, 81. 35633875 10.1016/j.bioactmat.2022.05.007 PMC9131252

[83] J. N. Tiwari , Y.‐K. Seo , T. Yoon , W. G. Lee , W. J. Cho , M. Yousuf , A. M. Harzandi , D.‐S. Kang , K.‐Y. Kim , P.‐G. Suh , K. S. Kim , ACS Nano 2017, 11, 742. 28033461 10.1021/acsnano.6b07138

[84] M. Schmelter , B. Ateghang , S. Helmig , M. Wartenberg , H. Sauer , FASEB J. 2006, 20, 1182. 16636108 10.1096/fj.05-4723fje

[85] Y. Sun , K. M. A. Yong , L. G. Villa‐Diaz , X. Zhang , W. Chen , R. Philson , S. Weng , H. Xu , P. H. Krebsbach , J. Fu , Nature Mater. 2014, 13, 599. 24728461 10.1038/nmat3945 PMC4051885

[86] J. H. Kim , H. W. Kim , K. J. Cha , J. Han , Y. J. Jang , D. S. Kim , J.‐H. Kim , ACS Nano 2016, 10, 3342. 26900863 10.1021/acsnano.5b06985

[87] L. Zhao , L. Liu , Z. Wu , Y. Zhang , P. K. Chu , Biomaterials 2012, 33, 2629. 22204980 10.1016/j.biomaterials.2011.12.024

[88] W. J. Hadden , J. L. Young , A. W. Holle , M. L. McFetridge , D. Y. Kim , P. Wijesinghe , H. Taylor‐Weiner , J. H. Wen , A. R. Lee , K. Bieback , B.‐N. Vo , D. D. Sampson , B. F. Kennedy , J. P. Spatz , A. J. Engler , Y. S. Choi , Proc. Natl. Acad. Sci. USA 2017, 114, 5647. 28507138 10.1073/pnas.1618239114 PMC5465928

[89] B. Coste , J. Mathur , M. Schmidt , T. J. Earley , S. Ranade , M. J. Petrus , A. E. Dubin , A. Patapoutian , Science 2010, 330, 55. 20813920 10.1126/science.1193270 PMC3062430

[90] X. Yang , C. Lin , X. Chen , S. Li , X. Li , B. Xiao , Nature 2022, 604, 377. 35388220 10.1038/s41586-022-04574-8

[91] J. Ge , W. Li , Q. Zhao , N. Li , M. Chen , P. Zhi , R. Li , N. Gao , B. Xiao , M. Yang , Nature 2015, 527, 64. 26390154 10.1038/nature15247

[92] L. He , G. Si , J. Huang , A. D. T. Samuel , N. Perrimon , Nature 2018, 555, 103. 29414942 10.1038/nature25744 PMC6101000

[93] B. S. Eftekhari , M. Eskandari , P. A. Janmey , A. Samadikuchaksaraei , M. Gholipourmalekabadi , Adv. Funct. Mater. 2020, 30, 1907792. 10.1039/d1ra00413a PMC9029165 35481217

[94] W. Zhang , Y. Yang , B. Cui , Curr. Opin. Solid State Mater. Sci. 2021, 25, 100873. 33364912 10.1016/j.cossms.2020.100873 PMC7751896

[95] H.‐W. Liu , W.‐C. Huang , C.‐S. Chiang , S.‐H. Hu , C.‐H. Liao , Y.‐Y. Chen , S.‐Y. Chen , Adv. Funct. Mater. 2014, 24, 3715.

[96] S. Park , G.‐I. Im , J. Biomed. Mater. Res. A 2015, 103, 1238. 24853234 10.1002/jbm.a.35236

[97] M. J. Dalby , N. Gadegaard , R. O. C. Oreffo , Nature Mater. 2014, 13, 558. 24845995 10.1038/nmat3980

[98] R. T. Moon , K. Shah , Nature 2002, 417, 239. 12015587 10.1038/417239a

[99] T. Pawson , Nature 1995, 373, 573. 7531822 10.1038/373573a0

[100] D. A. Fletcher , R. D. Mullins , Nature 2010, 463, 485. 20110992 10.1038/nature08908 PMC2851742

[101] Y. Sun , J. Liu , Z. Xu , X. Lin , X. Zhang , L. Li , Y. Li , Aging 2020, 13, 2231. 33318310 10.18632/aging.202244 PMC7880396

[102] A. Banerjee , M. Arha , S. Choudhary , R. S. Ashton , S. R. Bhatia , D. V. Schaffer , R. S. Kane , Biomaterials 2009, 30, 4695. 19539367 10.1016/j.biomaterials.2009.05.050 PMC2743317

[103] T.‐C. Tseng , L. Tao , F.‐Y. Hsieh , Y. Wei , I.‐M. Chiu , S. Hsu , Adv. Mater. 2015, 27, 3518. 25953204 10.1002/adma.201500762

[104] M. Li , P. Zhang , D. Zhang , Med. Hypotheses 2018, 114, 55. 29602466 10.1016/j.mehy.2018.02.027

[105] B. Sun , Y. Sun , S. Han , R. Zhang , X. Wang , C. Meng , T. Ji , C. Sun , N. Ren , S. Ge , H. Liu , Y. Yu , J. Wang , Int. J. Mol. Sci. 2023, 24, 530. 10.3390/ijms24010530 PMC9820130 36613973

[106] N. Adadi , M. Yadid , I. Gal , M. Asulin , R. Feiner , R. Edri , T. Dvir , Adv. Mater. Technol. 2020, 5, 1900820.

[107] E. Saburi , M. Islami , S. Hosseinzadeh , A. S. Moghadam , R. N. Mansour , E. Azadian , Z. Joneidi , A. R. Nikpoor , M. H. Ghadiani , Z. Khodaii , A. Ardeshirylajimi , Gene 2019, 696, 72. 30772518 10.1016/j.gene.2019.02.028

[108] R. F. Valentini , T. G. Vargo , J. A. Gardella , P. Aebischer , Biomaterials 1992, 13, 183. 1567943 10.1016/0142-9612(92)90069-z

[109] A. Bouaziz , A. Richert , A. Caprani , Biomaterials 1997, 18, 107. 9022957 10.1016/s0142-9612(96)00114-7

[110] N. Weber , Y.‐S. Lee , S. Shanmugasundaram , M. Jaffe , T. L. Arinzeh , Acta Biomater. 2010, 6, 3550. 20371302 10.1016/j.actbio.2010.03.035

[111] G. Xue , Y. Zhang , T. Xie , Z. Zhang , Q. Liu , X. Li , X. Gou , ACS Appl. Mater. Interfaces 2021, 13, 17361. 33823586 10.1021/acsami.1c02457

[112] H. Kawai , Jpn J Appl Phys. 1969, 8, 975.

[113] S. Han , D. Chen , J. Wang , Z. Liu , F. Liu , Y. Chen , Y. Ji , J. Pang , H. Liu , J. Wang , Nano Energy 2020, 72, 104688.

[114] Md. N. Islam , R. H. Rupom , P. R. Adhikari , Z. Demchuk , I. Popov , A. P. Sokolov , H. F. Wu , R. C. Advincula , N. Dahotre , Y. Jiang , W. Choi , Adv. Funct. Mater. 2023, 33, 2302946.

[115] H. H. Singh , N. Khare , Nano Energy 2018, 51, 216.

[116] R. Zhang , S. Han , L. Liang , Y. Chen , B. Sun , N. Liang , Z. Feng , H. Zhou , C. Sun , H. Liu , J. Wang , Nano Energy 2021, 87, 106192.

[117] L. Liang , C. Sun , R. Zhang , S. Han , J. Wang , N. Ren , H. Liu , Nano Energy 2021, 90, 106634.

[118] S. M. Damaraju , Y. Shen , E. Elele , B. Khusid , A. Eshghinejad , J. Li , M. Jaffe , T. L. Arinzeh , Biomaterials 2017, 149, 51. 28992510 10.1016/j.biomaterials.2017.09.024

[119] J. Liu , Y. Cheng , H. Wang , D. Yang , C. Liu , W. Dou , X. Jiang , H. Deng , R. Yang , Nano Energy 2023, 115, 108742.

[120] W.‐C. Chen , B.‐Y. Huang , S.‐M. Huang , S.‐M. Liu , K.‐C. Chang , C.‐L. Ko , C.‐L. Lin , Biomater. Adv. 2023, 144, 213228. 36481520 10.1016/j.bioadv.2022.213228

[121] V. Sencadas , C. Ribeiro , A. Heredia , I. K. Bdikin , A. L. Kholkin , S. Lanceros‐Mendez , Appl. Phys. A 2012, 109, 51.

[122] Y.‐H. Lai , Y.‐H. Chen , A. Pal , S.‐H. Chou , S.‐J. Chang , E.‐W. Huang , Z.‐H. Lin , S.‐Y. Chen , Nano Energy 2021, 90, 106545.

[123] M. Degli Esposti , F. Chiellini , F. Bondioli , D. Morselli , P. Fabbri , Mater. Sci. Eng. C 2019, 100, 286. 10.1016/j.msec.2019.03.014 30948063

[124] C. Ye , P. Hu , M.‐X. Ma , Y. Xiang , R.‐G. Liu , X.‐W. Shang , Biomaterials 2009, 30, 4401. 19481254 10.1016/j.biomaterials.2009.05.001

[125] Y. Zheng , L. Zhao , Y. Li , X. Zhang , W. Zhang , J. Wang , L. Liu , W. An , H. Jiao , C. Ma , Int. J. Mol. Sci. 2023, 24, 4051. 36835464 10.3390/ijms24044051 PMC9961896

[126] R. Mao , B. Yu , J. Cui , Z. Wang , X. Huang , H. Yu , K. Lin , S. G. F. Shen , Nano Energy 2022, 98, 107322.

[127] G. Ciofani , G. G. Genchi , V. Mattoli , Mater. Sci. Eng. C 2012, 32, 341.

[128] A. Blanquer , O. Careta , L. Anido‐Varela , A. Aranda , E. Ibáñez , J. Esteve , C. Nogués , G. Murillo , Int. J. Mol. Sci. 2022, 23, 432. 10.3390/ijms23010432 PMC8745485 35008860

[129] P. Yu , C. Ning , Y. Zhang , G. Tan , Z. Lin , S. Liu , X. Wang , H. Yang , K. Li , X. Yi , Y. Zhu , C. Mao , Theranostics 2017, 7, 3387. 28900517 10.7150/thno.19748 PMC5595139

[130] C. Shuai , G. Liu , Y. Yang , F. Qi , S. Peng , W. Yang , C. He , G. Wang , G. Qian , Nano Energy 2020, 74, 104825.

[131] G. G. Genchi , L. Ceseracciu , A. Marino , M. Labardi , S. Marras , F. Pignatelli , L. Bruschini , V. Mattoli , G. Ciofani , Adv. Healthcare Mater. 2016, 5, 1808. 10.1002/adhm.201600245 27283784

[132] J. Lei , C. Wang , X. Feng , L. Ma , X. Liu , Y. Luo , L. Tan , S. Wu , C. Yang , Chem. Eng. J. 2022, 435, 134624.

[133] T. Zheng , H. Zhao , Y. Huang , C. Gao , X. Zhang , Q. Cai , X. Yang , Ceram. Int. 2021, 47, 28778.

[134] Y. Li , X. Dai , Y. Bai , Y. Liu , Y. Wang , O. Liu , F. Yan , Z. Tang , X. Zhang , X. Deng , Int. J. Nanomed. 2017, 12, 4007. 10.2147/IJN.S135605 PMC5457183 28603415

[135] Z. Liu , M. Cai , X. Zhang , X. Yu , S. Wang , X. Wan , Z. L. Wang , L. Li , Adv. Mater. 2021, 33, 2106317. 10.1002/adma.202106317 34655105

[136] J.‐K. Yoon , T. I. Lee , S. H. Bhang , J.‐Y. Shin , J.‐M. Myoung , B.‐S. Kim , ACS Appl. Mater. Interfaces 2017, 9, 22101. 28560866 10.1021/acsami.7b03050

[137] W. Liu , Y. Luo , C. Ning , W. Zhang , Q. Zhang , H. Zou , C. Fu , J. Nanobiotechnology 2021, 19, 286. 34556136 10.1186/s12951-021-01031-y PMC8461877

[138] J. Du , G. Zhen , H. Chen , S. Zhang , L. Qing , X. Yang , G. Lee , H.‐Q. Mao , X. Jia , Biomaterials 2018, 181, 347. 30098570 10.1016/j.biomaterials.2018.07.015 PMC6201278

[139] T.‐Y. Park , J. Jeon , N. Lee , J. Kim , B. Song , J.‐H. Kim , S.‐K. Lee , D. Liu , Y. Cha , M. Kim , P. Leblanc , T. M. Herrington , B. S. Carter , J. S. Schweitzer , K.‐S. Kim , Nature 2023, 619, 606. 37438521 10.1038/s41586-023-06300-4 PMC12012854

[140] M. Colonna , O. Butovsky , Annu. Rev. Immunol. 2017, 35, 441. 28226226 10.1146/annurev-immunol-051116-052358 PMC8167938

[141] A. F. Lloyd , V. E. Miron , Nat. Rev. Neurol. 2019, 15, 447. 31256193 10.1038/s41582-019-0184-2

[142] A. Woodhoo , V. Sahni , J. Gilson , A. Setzu , R. J. M. Franklin , W. F. Blakemore , R. Mirsky , K. R. Jessen , Brain 2007, 130, 2175. 17550908 10.1093/brain/awm125

[143] S. A. G. Elsebay , H. Ali , H. Fikry , QJM. Int. J. Med. 2018, 111, hcy200.223.

[144] F. Féron , C. Perry , J. Cochrane , P. Licina , A. Nowitzke , S. Urquhart , T. Geraghty , A. Mackay‐Sim , Brain 2005, 128, 2951. 16219671 10.1093/brain/awh657

[145] X. Pan , W. Huang , G. Nie , C. Wang , H. Wang , Adv. Mater. 2023, 10.1002/adma.202303180. 37871967

[146] S. G. Varadarajan , J. L. Hunyara , N. R. Hamilton , A. L. Kolodkin , A. D. Huberman , Cell 2022, 185, 77. 34995518 10.1016/j.cell.2021.10.029 PMC10896592

[147] S. Temple , Cell Stem Cell 2023, 30, 512. 37084729 10.1016/j.stem.2023.03.017 PMC10201979

[148] L. Liu , J. Wu , S. Wang , L. Kun , J. Gao , B. Chen , Y. Ye , F. Wang , F. Tong , J. Jiang , J. Ou , D. A. Wilson , Y. Tu , F. Peng , Nano Lett. 2021, 21, 3518. 33848170 10.1021/acs.nanolett.1c00290

[149] X.‐Z. Chen , J.‐H. Liu , M. Dong , L. Müller , G. Chatzipirpiridis , C. Hu , A. Terzopoulou , H. Torlakcik , X. Wang , F. Mushtaq , J. Puigmartí‐Luis , Q.‐D. Shen , B. J. Nelson , S. Pané , Mater. Horiz. 2019, 6, 1512.

[150] Q. Zhang , B. Shi , J. Ding , L. Yan , J. P. Thawani , C. Fu , X. Chen , Acta Biomate. 2019, 88, 57. 10.1016/j.actbio.2019.01.056 30710714

[151] L. Li , B. Xiao , J. Mu , Y. Zhang , C. Zhang , H. Cao , R. Chen , H. K. Patra , B. Yang , S. Feng , Y. Tabata , N. K. H. Slater , J. Tang , Y. Shen , J. Gao , ACS Nano 2019, 13, 14283. 31769966 10.1021/acsnano.9b07598

[152] X. Ma , S. Zhukov , H. von Seggern , G. M. Sessler , O. Ben Dali , M. Kupnik , Y. Dai , P. He , X. Zhang , Adv. Electron. Mater. 2023, 9, 2201070.

[153] P. Chen , C. Xu , P. Wu , K. Liu , F. Chen , Y. Chen , H. Dai , Z. Luo , ACS Nano 2022, 16, 16513. 36174221 10.1021/acsnano.2c05818

[154] A. Zaszczynska , P. Sajkiewicz , A. Gradys , Polymers 2020, 12, 161.31936240

[155] M. Norouzi , S. M. Boroujeni , N. Omidvarkordshouli , M. Soleimani , Adv. Healthcare Mater. 2015, 4, 1114. 10.1002/adhm.201500001 25721694

[156] M. Tan , X. Xu , T. Yuan , X. Hou , J. Wang , Z. Jiang , L. Peng , Biomaterials 2022, 283, 121413. 35276616 10.1016/j.biomaterials.2022.121413

[157] W. A. Lackington , A. J. Ryan , F. J. O’Brien , ACS Biomater. Sci. Eng. 2017, 3, 1221. 33440511 10.1021/acsbiomaterials.6b00500

[158] H. Zhang , D. Lan , B. Wu , X. Chen , X. Li , Z. Li , F. Dai , Biomacromolecules 2023, 24, 3268. 37329512 10.1021/acs.biomac.3c00311

[159] C. Li , Y. Zhang , Y. Du , Z. Hou , Y. Zhang , W. Cui , W. Chen , Small Sci. 2023, 3, 2300027. 10.1002/smsc.202300027 PMC11935846 40213606

[160] A. C. Uzcategui , C. I. Higgins , J. E. Hergert , A. E. Tomaschke , V. Crespo‐Cuevas , V. L. Ferguson , S. J. Bryant , R. R. McLeod , J. P. Killgore , Small Sci. 2021, 1, 2000017. 34458889 10.1002/smsc.202000017 PMC8388578

[161] Y. Ma , H. Wang , Q. Wang , X. Cao , H. Gao , Chem. Eng. J. 2023, 452, 139424.

[162] W. Pi , F. Rao , J. Cao , M. Zhang , T. Chang , Y. Han , Y. Zheng , S. Liu , Q. Li , X. Sun , Y. Shao , Nano Today 2023, 50, 101860.

[163] Y. Liu , D. Luo , T. Wang , Small 2016, 12, 4611. 27322951 10.1002/smll.201600626

[164] L. Wang , Y. Pang , Y. Tang , X. Wang , D. Zhang , X. Zhang , Y. Yu , X. Yang , Q. Cai , Bioact. Mater. 2023, 25, 399. 37056250 10.1016/j.bioactmat.2022.11.004 PMC10087109

[165] R. Das , E. J. Curry , T. T. Le , G. Awale , Y. Liu , S. Li , J. Contreras , C. Bednarz , J. Millender , X. Xin , D. Rowe , S. Emadi , K. W. H. Lo , T. D. Nguyen , Nano Energy 2020, 76, 105028. 38074984 10.1016/j.nanoen.2020.105028 PMC10703347

[166] H. Liu , Y. Shi , Y. Zhu , P. Wu , Z. Deng , Q. Dong , M. Wu , L. Cai , ACS Appl. Mater. Interfaces 2023, 15, 12273. 36890691 10.1021/acsami.2c19767

[167] F. Zhao , C. Zhang , J. Liu , L. Liu , X. Cao , X. Chen , B. Lei , L. Shao , Chem. Eng. J 2020, 402, 126203.

[168] R. Dong , P. X. Ma , B. Guo , Biomaterials 2020, 229, 119584. 31704468 10.1016/j.biomaterials.2019.119584

[169] T. Tamaki , Y. Uchiyama , M. Hirata , H. Hashimoto , N. Nakajima , K. Saito , T. Terachi , J. Mochida , Front. Physiol. 2015, 6, 165. 26082721 10.3389/fphys.2015.00165 PMC4451695

[170] B. T. Corona , S. M. Greising , Biomaterials 2016, 104, 238. 27472161 10.1016/j.biomaterials.2016.07.020

[171] P. M. Martins , S. Ribeiro , C. Ribeiro , V. Sencadas , A. C. Gomes , F. M. Gama , S. Lanceros‐Méndez , RSC Adv. 2013, 3, 17938.

[172] J.‐K. Yoon , M. Misra , S. J. Yu , H. Y. Kim , S. H. Bhang , S. Y. Song , J.‐R. Lee , S. Ryu , Y. W. Choo , G.‐J. Jeong , S. P. Kwon , S. G. Im , T. I. Lee , B.‐S. Kim , Adv. Funct. Mater. 2017, 27, 1703853.

[173] S. Ribeiro , C. Ribeiro , E. O. Carvalho , C. R. Tubio , N. Castro , N. Pereira , V. Correia , A. C. Gomes , S. Lanceros‐Méndez , ACS Appl. Bio Mater. 2020, 3, 4239. 10.1021/acsabm.0c00315 35025425

[174] H. Liu , Y. Du , G. Yang , X. Hu , L. Wang , B. Liu , J. Wang , S. Zhang , Adv. Healthcare Mater. 2020, 9, 2000727. 10.1002/adhm.202000727 32743958

[175] J. Chen , L. Song , F. Qi , S. Qin , X. Yang , W. Xie , K. Gai , Y. Han , X. Zhang , Z. Zhu , H. Cai , X. Pei , Q. Wan , N. Chen , J. Wang , Q. Wang , Y. Li , Nano Energy 2023, 106, 108076.

[176] Y. Qian , Y. Xu , Z. Yan , Y. Jin , X. Chen , W.‐E. Yuan , C. Fan , Nano Energy 2021, 83, 105779.

[177] Y. Qian , Y. Cheng , J. Song , Y. Xu , W.‐E. Yuan , C. Fan , X. Zheng , Small 2020, 16, 2000796. 10.1002/smll.202000796 32633072

[178] A. Sharma , V. Panwar , B. Mondal , D. Prasher , M. K. Bera , J. Thomas , A. Kumar , N. Kamboj , D. Mandal , D. Ghosh , Nano Energy 2022, 99, 107419.

[179] C. Li , S. Zhang , Y. Yao , Y. Wang , C. Xiao , B. Yang , J. Huang , W. Li , C. Ning , J. Zhai , P. Yu , Y. Wang , Adv. Healthcare Mater. 2023, 12, 2300064. 10.1002/adhm.202300064 36854114

[180] T. S. Pinho , D. Silva , J. C. Ribeiro , A. Marote , R. Lima , S. J. Batista , R. Melo , C. Ribeiro , C. B. Cunha , I. S. Moreira , S. Lanceros‐Mendez , A. J. Salgado , J. Biomed. Mater. Res. A 2023, 111, 35. 36069387 10.1002/jbm.a.37443

[181] G. Xia , G. Wang , H. Yang , W. Wang , J. Fang , Nano Energy 2022, 102, 107690.

[182] G. Li , Z. Li , Y. Min , S. Chen , R. Han , Z. Zhao , Small 2023, 19, 2302927. 10.1002/smll.202302927 37264732

[183] Z. Zhou , P. Yu , L. Zhou , L. Tu , L. Fan , F. Zhang , C. Dai , Y. Liu , C. Ning , J. Du , G. Tan , ACS Biomater. Sci. Eng. 2019, 5, 4386. 33438404 10.1021/acsbiomaterials.9b00812

[184] B. Ma , F. Liu , Z. Li , J. Duan , Y. Kong , M. Hao , S. Ge , H. Jiang , H. Liu , J. Mater. Chem. B 2019, 7, 1847. 32255047 10.1039/c8tb03321h

[185] K. Cai , Y. Jiao , Q. Quan , Y. Hao , J. Liu , L. Wu , Bioact. Mater. 2021, 6, 4073. 33997494 10.1016/j.bioactmat.2021.04.016 PMC8090998

[186] V. K. Kaliannagounder , N. P. M. J. Raj , A. R. Unnithan , J. Park , S. S. Park , S.‐J. Kim , C. H. Park , C. S. Kim , A. R. K. Sasikala , Nano Energy 2021, 85, 105901.

